# Pneumococcal vaccine uptake among high-risk adults and children in Italy: results from the OBVIOUS project survey

**DOI:** 10.1186/s12889-024-18216-3

**Published:** 2024-03-07

**Authors:** Zeno Di Valerio, Giusy La Fauci, Francesca Scognamiglio, Aurelia Salussolia, Marco Montalti, Angelo Capodici, Maria Pia Fantini, Anna Odone, Claudio Costantino, Giorgia Soldà, Heidi J. Larson, Julie Leask, Jacopo Lenzi, Davide Gori, the OBVIOUS board, Angelo Capodici, Michele Conversano, Claudio Costantino, Mirko Degli Esposti, Zeno Di Valerio, Maria Pia Fantini, Davide Gori, Andrea Grignolio, Giusy La Fauci, Heidi Larson, Julie Leask, Jacopo Lenzi, Marco Montalti, Anna Odone, Daniel Remondini, Francesca Scognamiglio, Aurelia Salussolia, Giorgia Soldà, Federico Toth, Francesco Vitale

**Affiliations:** 1 Department of Biomedical and Neuromotor Sciences, Unit of Hygiene, Public Health and Medical Statistics, University of Bologna, 40126 Bologna, Italy; 2 Department of Public Health, Experimental and Forensic Medicine, University of Pavia, Pavia, Italy; 3 Department of Health Promotion Sciences, Maternal and Infant Care, Internal Medicine and Excellence Specialties “G. D’Alessandro”, University of Palermo, Palermo, Italy; 4 London School of Hygiene and Tropical Medicine (LSHTM), London, UK; 5 University of Washington, Seattle, WA USA; 6 School of Public Health, Faculty of Medicine and Health, University of Sydney, Sydney, NSW Australia

**Keywords:** Pneumococcal vaccine, Vaccine uptake, Immunization

## Abstract

**Background:**

*Streptococcus pneumoniae* infections, including Invasive Pneumococcal Diseases (IPDs), pose a substantial public health challenge, causing significant morbidity and mortality, especially among children and older adults. Vaccination campaigns have played a vital role in reducing pneumococcal-related deaths. However, obstacles related to accessibility and awareness might impede optimal vaccine adoption. This study aims to provide comprehensive data on pneumococcal vaccine coverage and attitudes within at-risk groups in Italy, with the goal of informing public health strategies and addressing vaccination barriers.

**Methods:**

Between April 11 and May 29, 2022, a questionnaire investigating vaccine uptake and attitudes toward several vaccinations was administered to 10,000 Italian adults, chosen through population-based sampling. Respondents who were targets of the campaign according to the 2017–2019 National Vaccination Plan, accessed questions regarding pneumococcal vaccination. Data on uptake, awareness of having the right to free vaccination, opinion on vaccine safety, concern with pneumococcal disease, and ease of access to vaccination services were summarized and presented based on statistical regions. Multinomial logistic regression analysis was used to explore factors influencing vaccine uptake.

**Results:**

Out of 2357 eligible adult respondents (42.6% women; mean age: 58.1 ± 15.7), 39.5% received pneumococcal vaccination. Uptake differed among at-risk groups: respondents aged ≥65 (33.7%), with lung disease (48.4%), cardiovascular disease (46.6%), and diabetes (53.7%). Predictors of not being vaccinated and unwilling to included female gender, residing in rural areas, lower education, low concern about pneumococcal disease, vaccine safety concerns, and associations with vaccine-opposed acquaintances. Health access issues predicted willingness to be vaccinated despite non-vaccination. Pneumopathy, heart disease, diabetes, and living in Northeastern or Central Italy were linked to higher uptake. Among the 1064 parents of eligible children, uptake was 79.1%. Parental unawareness of children’s free vaccination eligibility was a predictor of non-vaccination. Vaccine safety concerns correlated with reluctance to vaccinate children, while perceived healthcare access challenges were associated with wanting but not having received vaccination.

**Conclusions:**

Pneumococcal vaccination uptake within prioritized groups and children in Italy remains inadequate. Scarce awareness of vaccine availability and obstacles in accessing vaccinations emerge as principal barriers influencing this scenario.

**Supplementary Information:**

The online version contains supplementary material available at 10.1186/s12889-024-18216-3.

## Background

*Streptococcus pneumoniae* are commonly found in the respiratory tracts of healthy individuals, particularly children, and are a frequent cause of infections such as otitis media, sinusitis, conjunctivitis, and community-acquired pneumonia. In certain conditions, pneumococci can cause more serious conditions known collectively as Invasive Pneumococcal Diseases (IPDs), which include meningitis, sepsis, and osteomyelitis [1]. Before the introduction of the first conjugated vaccine in 2001, it is estimated that *S. pneumoniae* infections caused more than 108,000 deaths each year in the European Union, accounting for about 2.2% of all deaths. It also caused 5.3% of deaths in children younger than 5 and about 65% of deaths due to lower respiratory tract infections in adults older than 55 [2]. In Italy, 1680 cases of invasive pneumococcal disease were reported in 2019 with an incidence of 2.81 cases per 100,000 population. The most frequent forms of IPD during the 2019–2021 period were sepsis/bacteremia (37–55% of cases), pneumonia with sepsis/bacteremia (25–36% of cases), and meningitis (18–25% of cases). Notably, children and older adults bore the brunt of the burden [3]. The serious death toll and burden on health services caused by pneumococcal infections and IPD prompted early attempts at developing an effective vaccine. Currently, virtually all European countries have implemented vaccination campaigns [4–6]. There are more than 90 known serotypes of *S. pneumoniae*, with varying distributions across geographic areas and populations. However, a small number of these serotypes are responsible for the majority of IPD cases, making them the primary target of preventive efforts [7]. Two types of vaccines are currently in use: polysaccharide vaccines and conjugate vaccines. Polysaccharide vaccines, which contain the purified capsular antigen, were the first to be devised and marketed in the early 80’s. They include PPV-23, which is still in use in most European countries today [6, 8]. Conjugate vaccines are constituted by a carrier protein bound to the capsule polysaccharide; many have been approved and replaced by newer ones protecting against a wider array of serotypes, including PCV-7, PCV-10 and PCV-13, and the more recently approved PCV-15 and PCV-20 [9–12]. Since their introduction, pneumococcal vaccines have had a profound impact on the epidemiology of IPDs and other related infections, leading to a decline in pneumococcal disease mortality rates across all demographics in Europe and Italy, and to shifts in serotype prevalence in areas where vaccination campaigns were implemented [2]. In adults, PPV-23 has played and is still playing a significant role in this respect, although its public health impact has been limited by its inefficacy in children under 2 years old and its inability to generate a lasting T-cell immune response. In fact, its usefulness may have diminished since its introduction, likely due to shifts in serotype prevalence over the last three decades, in response to a selective advantage of serotypes not included in the original 23-valent formulation [6, 13]. Immunizing children with PCV-7, which received approval for use in Europe in 2001 and has been included in children’s vaccination schedules in many European countries, has been shown to reduce IPD cases caused by vaccine serotypes by 80% in a large Danish cohort study [14]. Furthermore, in the USA, overall IPD incidence declined by 45%, and PCV-7-type IPD cases decreased by an impressive 94% [15]. Similar results were observed with later conjugated vaccines, such as PCV-10/13 [6]. In Italy, pneumococcal vaccination is currently actively offered free of charge to newborns in their first year, children up to 5 years old, and individuals aged 65. Additionally, some specific health conditions also grant free access to vaccination regardless of age: chronic heart disease, chronic lung disease, diabetes mellitus, chronic liver disease, cerebrospinal fluid leaks, cochlear implants, hemoglobinopathies, immunodeficiencies, asplenia, oncologic and hematologic disorders, organ or bone marrow transplantation, conditions requiring long-term immunosuppressive treatment, and chronic kidney disease or adrenal insufficiency. Vaccination schedules, in accordance with 2017–2019 National Immunization Plan, for newborns recommend the use of a conjugated vaccine and consist of three doses administered during the third, fifth, and eleventh month of age. For 65-year-olds and other at-risk individuals, the recommended schedule includes administration of a conjugated vaccine followed by a polysaccharide vaccine in this order [16]. Despite evidence backing the need for pneumococcal vaccination campaigns, their effectiveness is impaired by limited knowledge about the right to free vaccination and other barriers in obtaining the vaccine, including willingness to get vaccinated. The current lack of publicly accessible official and disaggregated data on pneumococcal vaccination coverage in Italian at-risk adults and children, coupled with limited literature on the subject, drove us to conduct this survey. We expect our data on uptake and attitudes towards vaccination to be of assistance to decision-makers in public health for assessing the effectiveness of Italy’s vaccination campaign and directing efforts towards low-uptake demographics and key barriers to vaccination.

## Methods

### Study design

We conducted a cross-sectional study with the objective of evaluating vaccination uptake and exploring the attitudes of Italian citizens towards five vaccines provided free of charge by the National Health Service to specific target population categories. Specifically, our investigation covered vaccines for Rotavirus, Pneumococcus, Human Papillomavirus, Influenza, and Herpes Zoster Virus. To gather data, we administered a comprehensive 62-question questionnaire to a sample of 10,000 citizens, selected to represent the Italian population in terms of age, gender and region of residence. The study presented here focuses on assessing vaccination uptake, attitudes towards vaccination, and factors influencing the decision-making process related to the pneumococcal vaccine.

### Questionnaire

We designed a 62-question questionnaire by adapting the WHO Behavioural and Social Drivers (BeSD) of vaccination conceptual framework [17]. Survey items were informed by those in the WHO/UNICEF BeSD guidance but not identical as the definitive items were not available when the OBVIOUS survey was developed [18]. The questionnaire was designed to be completed in approximately 10 minutes and consisted of seven different sections. Section 1 investigated demographic characteristics; section 2 to 6 investigated vaccination uptake and attitudes towards the five vaccines among vaccination’s population targets; section 7 investigated COVID-19 vaccination status, attitudes towards different policy tools, and use of alternative medicine. All respondents had access to the first and the last sections of the questionnaire, while sections 2 to 6 were specifically delivered to individuals who fell under the vaccination’s population targets as defined by the 2017–2019 National Vaccination Plan, based on specific requirements such as age group, gender, medical history, body mass index (BMI), and profession [16]. The section pertaining to pneumococcal vaccination was accessed by individuals suffering from chronic heart disease, chronic lung disease and/or diabetes, and by individuals aged 65 and above. Additionally, the section was accessed by parents whose youngest child was between 9 weeks and 9 years old: the choice of only investigating vaccination status of the youngest child was made to limit recall bias and questionnaire length. Wording of the questions was adjusted accordingly. Informed consent was requested at the beginning of the questionnaire; if consent was not granted, the questionnaire was terminated. A translated English version of the questionnaire is available in the supplementary material (Additional file 1).

### Data collection

The questionnaire was administered between 11 April and 29 May, 2022 using computer-assisted web interviewing (CAWI), a surveying method that allows for the autonomous completion of surveys through purposefully built web applications. The professional online panel provider Dynata recruited a national sample of 10,000 respondents among its large proprietary panel of Italian residents. A stratified sampling method was used based on proportionate allocation by first-level NUTS statistical region of residence (Northwest, Northeast, Center, South, and Islands), gender, and age group (18–24, 25–34, 35–44, 45–54, 55–64, and ≥ 65 years). Post-stratification confirmed that non-response to the survey in some strata of Italy’s adult target population had no substantial effect on the study estimates [19]. For this reason, we deemed it as unnecessary to adjust sampling weights on the target subsample of respondents for pneumococcal vaccination (*n* = 3910). Data management by Dynata for the survey was conducted in compliance with the General Data Protection Regulation (GDPR) of the European Union. The GDPR ensures the protection and privacy of personal data of individuals within the European Union. Furthermore, the survey adhered to all relevant requirements outlined by Italian regulations, ensuring that data collection, storage, and processing were conducted in accordance with applicable laws and guidelines in Italy. These measures were implemented to safeguard the privacy and confidentiality of participants’ information throughout the survey process.

### Statistical analysis

All variables were summarized as counts and percentages and were stratified by first-level NUTS (Nomenclature of Territorial Units for Statistics) statistical region of residence and by target group based on age, gender, and clinical history (male vs. female adults, children, ages ≥65 years, and individuals with pneumopathy, heart disease or diabetes). Data were visualized with the aid of square charts and thematic maps with pie charts. Square charts, also called waffle charts, are a form of pie charts that use 10 × 10 grids instead of circles to represent percentages. Multivariable multinomial logistic regression analysis was carried out to examine the drivers (determinants) of vaccine uptake, which was considered as a three-category nominal outcome (“I did get the vaccine” vs. “I did not get the vaccine, but I would” vs. “I do not want to get vaccinated”). In keeping with the increasing vaccination model proposed by the BeSD Expert Working Group [17], the covariates included in the regression model as potential drivers of vaccine hesitancy were the following: thoughts and beliefs about pneumococcal infection and vaccination (perceived worry and safety concerns), social processes (friends and family’s views on vaccination, gender), and practical issues (awareness of having higher priority for vaccination, and perceived ease of access to healthcare to get the vaccine). Relevant sociodemographic determinants were also considered (age group, statistical region of residence, place of residence, degree of urbanization, and educational attainment), as well as clinical factors that lead to a higher priority for vaccination (pneumopathy, heart disease, and diabetes). Contrary to the BeSD theoretical model, the latent concept of “motivation” could not be tested as a mediator in the association of beliefs and social processes with vaccine uptake, because in our cross-sectional design vaccine uptake and motivation were collected in one solution, leading to three mutually exclusive outcome categories—in statistical terms, those unwilling to get vaccinated did not get the vaccine by definition. The effect of covariates was assessed by examining the marginal effect of changing their values on the average predicted probability of observing each outcome. The marginal effect was computed as a discrete difference in probabilities (Δ), with 95% confidence intervals (CIs) obtained with the delta method. Covariate categories occurring in < 5% of the sample were combined with adjacent lower or upper classes to improve the stability and efficiency of regression estimates. The Small–Hsiao test of independence of irrelevant alternatives (IIA) did not indicate the need for alternative model specifications in which binary logit coefficients do not converge in probability to the same values as the multinomial logit coefficients, such as the nested logit model. Lastly, in order to check for the presence of moderators, that is, covariates *Z* that change the effect of other independent variables *X* on vaccine uptake, we included pairwise interaction terms *Z* × *X* in the model one at a time and tested their statistical significance with the likelihood-ratio (LR) test. To control for type I error related to multiple testing, the significance level for interactions was set at 0.01. All analyses were conducted using Stata software, version 17 (StataCorp. 2021. Stata Statistical Software: Release 17. College Station, TX: StataCorp LP), and were performed separately on individuals answering on their own behalf vs. individuals answering on their children’s behalf. No multicollinearity issues were found in regression analysis, that is, the variance inflation factor was < 5 and the condition index was < 10 for each covariate.

## Results

Overall, 3910 respondents from the main sample of 10,000 met the criteria for accessing the section regarding pneumococcal vaccination: among these, 2677 (68.5%) answered on their own behalf and 1233 (31.5%) on behalf of their youngest child. Following the exclusion of 489 participants (12.5%) who were unable to recall their own or their children’s vaccination status, the analysis encompassed a final sample size of 3421 individuals, of which 2357 (68.9%) answered on their own behalf and 1064 (31.1%) on their children’s behalf.

### Adults

**Fig. 1:**
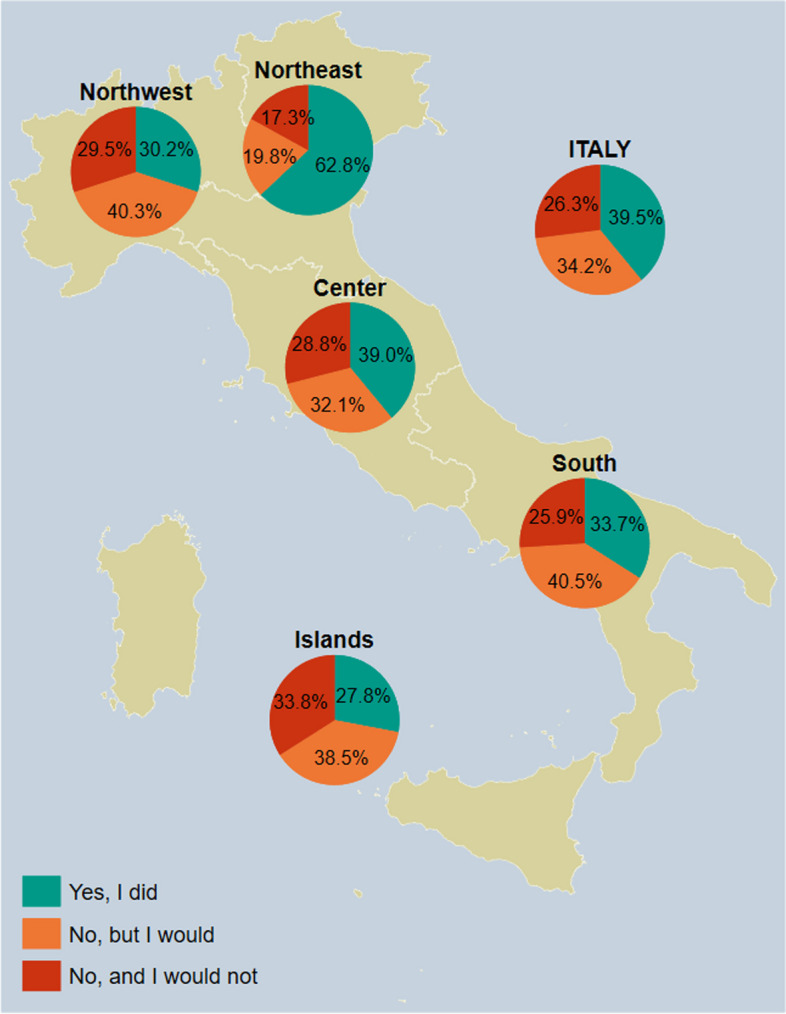
Pneumococcal vaccine uptake among respondents who answered on their own behalf (*n* = 2357), overall and by NUTS statistical region; if the answer is no, the respondents are asked whether or not they would get the vaccine. *Notes:* Northwestern Italy includes the regions of Piedmont, Aosta Valley, Lombardy, and Liguria; Northeastern Italy includes the regions of Trentino-South Tyrol, Veneto, Friuli-Venezia Giulia, and Emilia-Romagna; Central Italy includes the regions of Tuscany, Umbria, Marche, and Lazio; Southern Italy includes the regions of Abruzzo, Molise, Campania, Apulia, Basilicata, and Calabria; Insular Italy includes the regions of Sicily and Sardinia. *NUTS*, Nomenclature of Territorial Units for Statistics

**Fig. 2:**
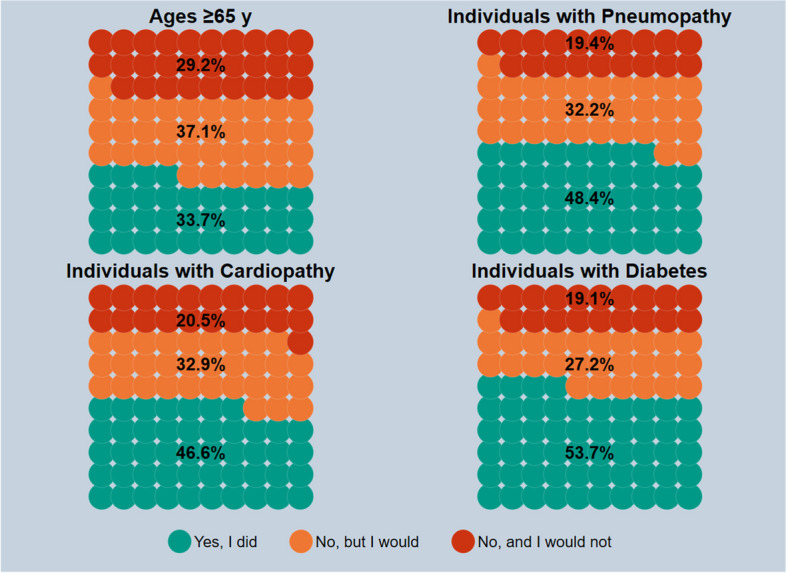
Pneumococcal vaccine uptake among respondents who answered on their own behalf, by high-risk target group based on age or clinical status; if the answer is no, the respondents are asked whether or not they would get the vaccine

**Fig. 3:**
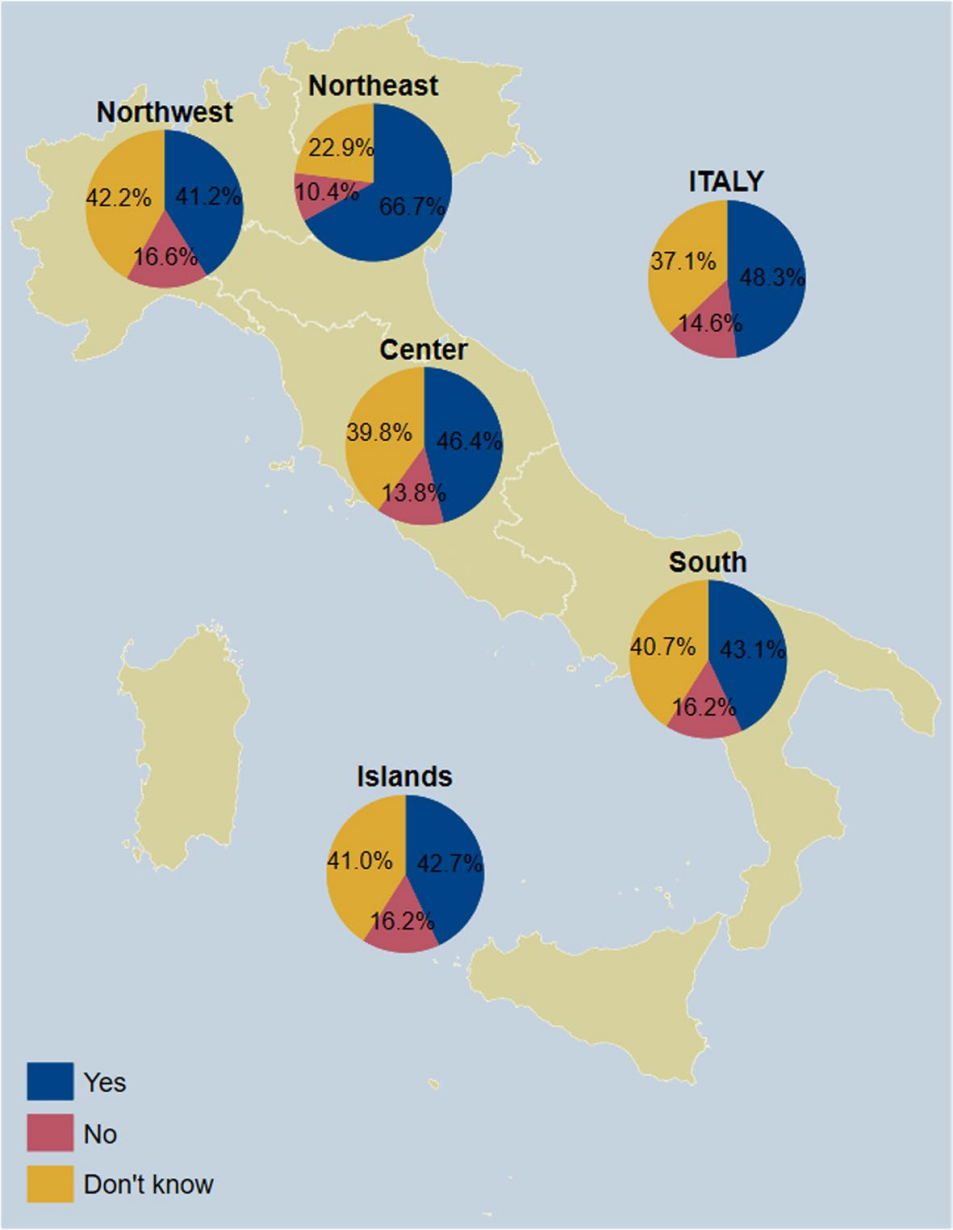
Awareness of having higher priority for pneumococcal vaccination among respondents who answered on their own behalf (*n* = 2357), overall and by NUTS statistical region. *Notes:* Northwestern Italy includes the regions of Piedmont, Aosta Valley, Lombardy, and Liguria; Northeastern Italy includes the regions of Trentino-South Tyrol, Veneto, Friuli-Venezia Giulia, and Emilia-Romagna; Central Italy includes the regions of Tuscany, Umbria, Marche, and Lazio; Southern Italy includes the regions of Abruzzo, Molise, Campania, Apulia, Basilicata, and Calabria; Insular Italy includes the regions of Sicily and Sardinia. *NUTS*, Nomenclature of Territorial Units for Statistics

**Fig. 4:**
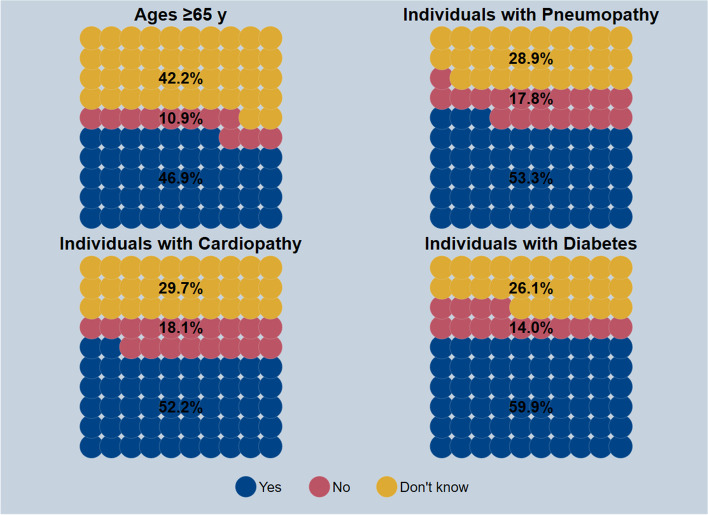
Awareness of having higher priority for pneumococcal vaccination among respondents who answered on their own behalf, by high-risk target group based on age or clinical status

**Fig. 5:**
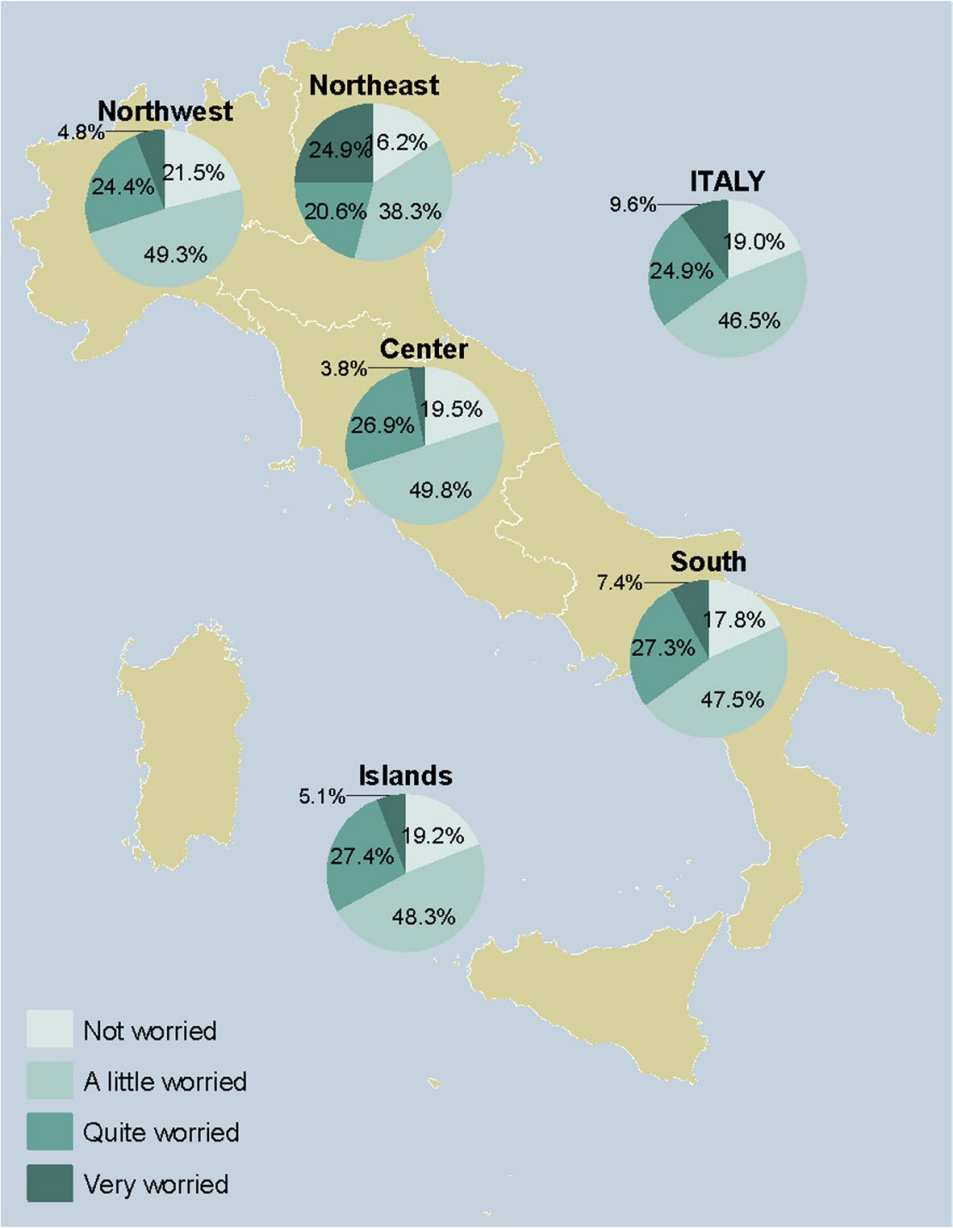
Worry about getting sick with pneumococcal pneumonia among respondents who answered on their own behalf (*n* = 2357), overall and by NUTS statistical region. *Notes:* Northwestern Italy includes the regions of Piedmont, Aosta Valley, Lombardy, and Liguria; Northeastern Italy includes the regions of Trentino-South Tyrol, Veneto, Friuli-Venezia Giulia, and Emilia-Romagna; Central Italy includes the regions of Tuscany, Umbria, Marche, and Lazio; Southern Italy includes the regions of Abruzzo, Molise, Campania, Apulia, Basilicata, and Calabria; Insular Italy includes the regions of Sicily and Sardinia. *NUTS*, Nomenclature of Territorial Units for Statistics

**Fig. 6:**
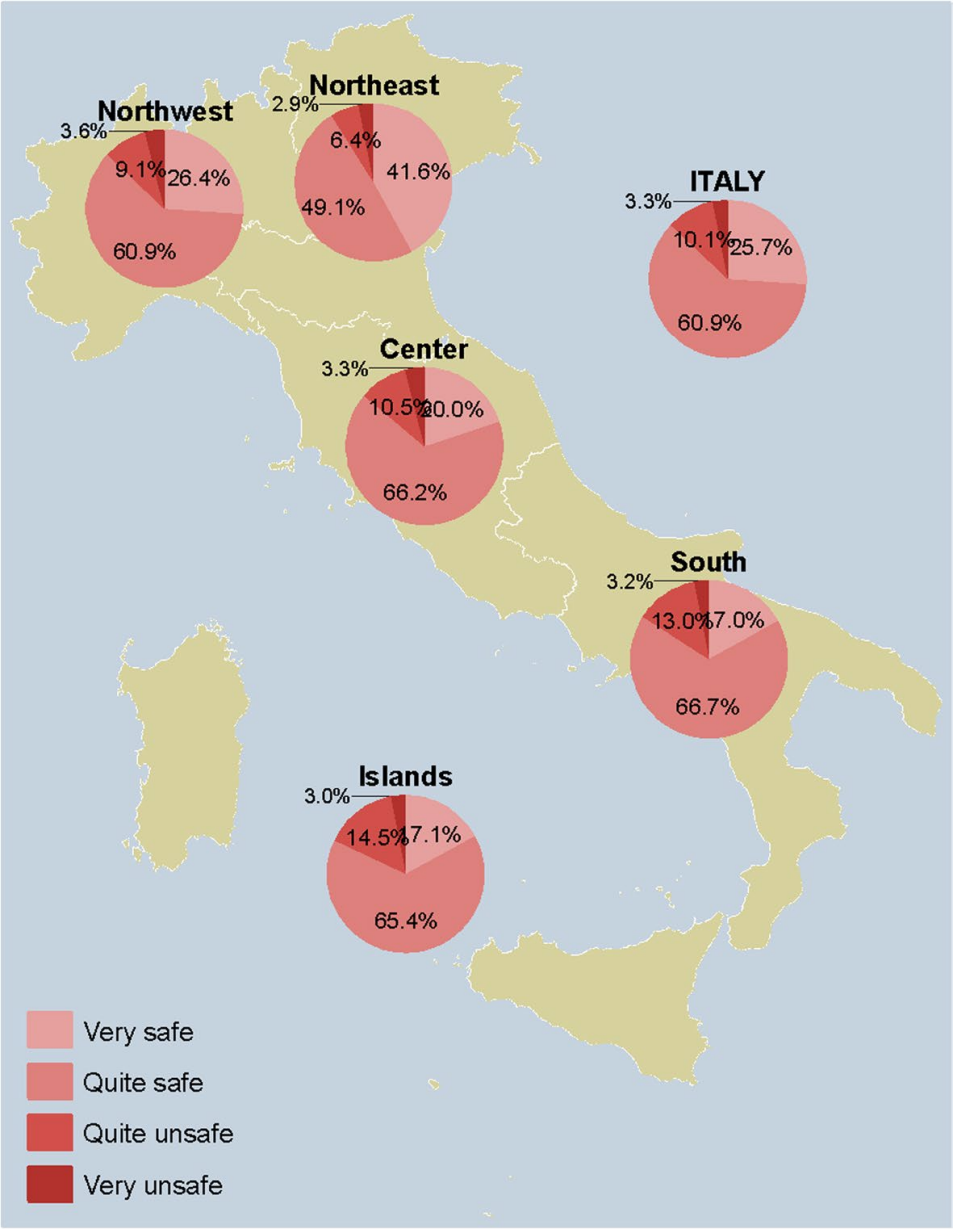
Perception of the safety of pneumococcal vaccine among respondents who answered on their own behalf (*n* = 2357), overall and by NUTS statistical region. *Notes:* Northwestern Italy includes the regions of Piedmont, Aosta Valley, Lombardy, and Liguria; Northeastern Italy includes the regions of Trentino-South Tyrol, Veneto, Friuli-Venezia Giulia, and Emilia-Romagna; Central Italy includes the regions of Tuscany, Umbria, Marche, and Lazio; Southern Italy includes the regions of Abruzzo, Molise, Campania, Apulia, Basilicata, and Calabria; Insular Italy includes the regions of Sicily and Sardinia. *NUTS*, Nomenclature of Territorial Units for Statistics

**Fig. 7:**
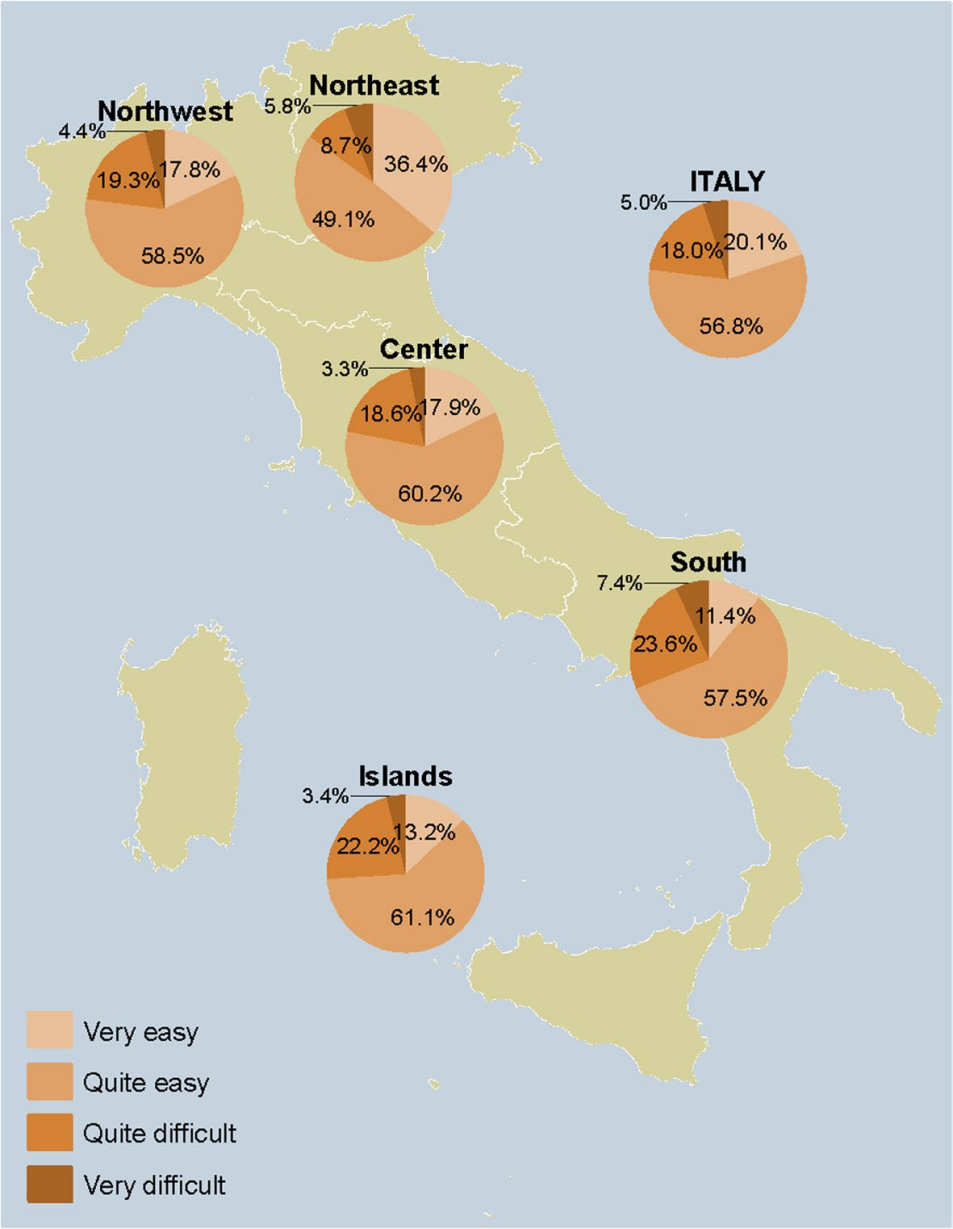
Perception of how easy it is to access healthcare facilities to get a pneumococcal vaccine among respondents who answered on their own behalf (*n* = 2357), overall and by NUTS statistical region. *Notes:* Northwestern Italy includes the regions of Piedmont, Aosta Valley, Lombardy, and Liguria; Northeastern Italy includes the regions of Trentino-South Tyrol, Veneto, Friuli-Venezia Giulia, and Emilia-Romagna; Central Italy includes the regions of Tuscany, Umbria, Marche, and Lazio; Southern Italy includes the regions of Abruzzo, Molise, Campania, Apulia, Basilicata, and Calabria; Insular Italy includes the regions of Sicily and Sardinia. *NUTS*, Nomenclature of Territorial Units for Statistics

Among the 2357 respondents who answered on their own behalf, 42.6% were women and mean age was 58.1 ± 15.7. NUTS geographical area of residence was Northwestern Italy for 685 respondents (29.1%), Northeastern Italy for 519 (22.0%), Central Italy for 420 (17.8%), South Italy for 499 (21.2%), and Insular Italy for 234 (9.9%). A total of 575 (24.4%) declared suffering from some form of cardiovascular disease, 516 (21.9%) from lung disease and 750 (31.8%) from diabetes, while 403 (17.1%) had BMI equal or higher than 30 (Table 1). Overall, 930 (39.5%) had received some kind of pneumococcal vaccination, 621 (26.3%) had not and would not want to receive any, and 806 (34.2%) had not but would want to. The highest uptake was registered in the Northeast, at 62.8%, while the lowest was registered in the Islands, at 27.8% (Fig. 1). Data on uptake for each region of Italy can be found in the supplementary material (Additional file 2). Stratifying by gender, we found an overall uptake of 33.4% among females and 44.0% among males. In the prioritized at-risk groups identified by the national vaccination campaign, the uptake of the vaccine was found to be 33.7% among respondents aged ≥65, 48.4% among individuals with lung disease, 46.6% among individuals with cardiovascular disease, and 53.7% among individuals with diabetes (Fig. 2). With regard to awareness of being a target of the national vaccination campaign, 1.138 respondents (48.3%) believed that they were among the at-risk groups that have the right to pneumococcal vaccination free of charge, while 345 (14.6%) believed they were not and 874 (37.1%) did not know. The highest proportion of respondents that believed they had the right to free vaccination was registered in the Northeast, at 66.7%, while the lowest proportion was registered in the Northwest, at 41.2% (Fig. 3). Stratifying by gender, we found an awareness of 44.3% among females and 51.3% among males. Among the at-risk groups prioritized by the national vaccination campaign, awareness was 46.9% among respondents aged ≥65, 53.3% among individuals with lung disease, 52.2% among individuals with cardiovascular disease, and 59.9% among individuals with diabetes (Fig. 4). Regarding risk perception, 227 respondents (9.6%) were very worried about pneumococcal disease, 587 (24.9%) were quite worried, 1096 (46.5%) were a little worried, and 447 (19.0%) were not worried. The highest proportion of respondents who were quite worried or very worried was registered in the Northeast, at 45.5%, while the lowest proportion was registered in the Northwest, at 29.2% (Fig. 5). Stratifying by gender, we found that 33.7% of females and 35.2% of males were quite worried or very worried about pneumococcal disease. In the at-risk groups prioritized by the national vaccination campaign, 26.8% were quite worried or very worried about pneumococcal disease among respondents aged ≥65, 46.9% among individuals with lung disease, 40.9% among individuals with cardiovascular disease, and 46.4% among individuals with diabetes. Overall, 606 respondents (25.7%) thought that the vaccine was very safe, 1436 (60.9%) quite safe, 238 (10.1%) quite unsafe, and 77 (3.3%) very unsafe. The highest proportion of respondents who viewed the vaccine as quite or very safe was registered in the Northeast, at 90.7%, while the lowest proportion was registered in the Islands, at 72.5% (Fig. 6). Stratifying by gender, we found that 85.5% of females and 87.5% of males viewed the vaccine as quite or very safe. Among the at-risk groups prioritized by the national vaccination campaign, 88.6% thought the vaccine was quite or very safe among respondents aged ≥65, 79.9% among individuals with lung disease, 83.8% among individuals with cardiovascular disease, and 85.2% among individuals with diabetes. When considering perceived ease of access to vaccination, 474 respondents (20.1%) found it very easy to access vaccination, 1339 (56.8%) quite easy, 425 (18.0%) quite difficult, and 119 (5.0%) very difficult. The highest proportion of respondents who viewed access as very or quite easy was registered in the Northeast, at 85.5%, while the lowest proportion was registered in the Islands, at 68.9% (Fig. 7). Stratifying by gender, we found that 73.3% of females and 79.4% of males viewed access as very or quite easy. Among the at-risk groups prioritized by the national vaccination campaign, 83.0% found access to vaccination to be very easy or quite easy among respondents aged ≥65, 63.0% among individuals with lung disease, 69.3% among individuals with cardiovascular disease, and 73.2% among individuals with diabetes. More data on attitudes of vulnerable adults by risk condition and gender can be found in the supplementary material (Additional files 3, 4, 5, 6).

**Table 1:** Sociodemographic and clinical characteristics of the study respondents who provided information about their own pneumococcal vaccine uptake, overall and by NUTS statistical region

Characteristic	Italy	Northwestern Italy	Northeastern Italy	Central Italy	Southern Italy	Insular Italy
	(*n* = 2357)	(*n* = 685)	(*n* = 519)	(*n* = 420)	(*n* = 499)	(*n* = 234)
Gender						
Male	1344 (57.0%)	374 (54.6%)	343 (66.1%)	235 (56.0%)	275 (55.1%)	117 (50.0%)
Female	1005 (42.6%)	309 (45.1%)	175 (33.7%)	185 (44.0%)	221 (44.3%)	115 (49.1%)
Non-binary	8 (0.3%)	2 (0.3%)	1 (0.2%)	0 (0.0%)	3 (0.6%)	2 (0.9%)
Age group, y						
18–24	68 (2.9%)	16 (2.3%)	14 (2.7%)	13 (3.1%)	18 (3.6%)	7 (3.0%)
25–34	186 (7.9%)	37 (5.4%)	52 (10.0%)	30 (7.1%)	49 (9.8%)	18 (7.7%)
35–44	282 (12.0%)	75 (10.9%)	93 (17.9%)	36 (8.6%)	47 (9.4%)	31 (13.2%)
45–54	249 (10.6%)	68 (9.9%)	61 (11.8%)	42 (10.0%)	50 (10.0%)	28 (12.0%)
55–64	251 (10.6%)	72 (10.5%)	43 (8.3%)	53 (12.6%)	58 (11.6%)	25 (10.7%)
≥ 65	1321 (56.0%)	417 (60.9%)	256 (49.3%)	246 (58.6%)	277 (55.5%)	125 (53.4%)
Place of residence degree of urbanization*						
City (densely populated area)	933 (39.6%)	308 (45.0%)	127 (24.5%)	167 (39.8%)	249 (49.9%)	82 (35.0%)
Town or suburb (intermediate density area)	1025 (43.5%)	304 (44.4%)	191 (36.8%)	195 (46.4%)	204 (40.9%)	131 (56.0%)
Rural area (thinly populated area)	399 (16.9%)	73 (10.7%)	201 (38.7%)	58 (13.8%)	46 (9.2%)	21 (9.0%)
Educational attainment						
Less than high school diploma	341 (14.5%)	113 (16.5%)	72 (13.9%)	53 (12.6%)	58 (11.6%)	45 (19.2%)
High school diploma	1253 (53.2%)	399 (58.2%)	210 (40.5%)	241 (57.4%)	280 (56.1%)	123 (52.6%)
Academic degree	459 (19.5%)	121 (17.7%)	87 (16.8%)	91 (21.7%)	113 (22.6%)	47 (20.1%)
Post-graduate/Doctorate degree	304 (12.9%)	52 (7.6%)	150 (28.9%)	35 (8.3%)	48 (9.6%)	19 (8.1%)
Household composition						
Alone	358 (15.2%)	134 (19.6%)	74 (14.3%)	61 (14.5%)	60 (12.0%)	29 (12.4%)
Couple	1653 (70.1%)	461 (67.3%)	388 (74.8%)	301 (71.7%)	348 (69.7%)	155 (66.2%)
With parents/family	227 (9.6%)	59 (8.6%)	37 (7.1%)	30 (7.1%)	68 (13.6%)	33 (14.1%)
Other	119 (5.0%)	31 (4.5%)	20 (3.9%)	28 (6.7%)	23 (4.6%)	17 (7.3%)
Able to pay for things needed in life						
With great difficulty	340 (14.4%)	86 (12.6%)	45 (8.7%)	68 (16.2%)	99 (19.8%)	42 (17.9%)
With some difficulty	990 (42.0%)	277 (40.4%)	163 (31.4%)	195 (46.4%)	228 (45.7%)	127 (54.3%)
Quite easily	824 (35.0%)	273 (39.9%)	194 (37.4%)	144 (34.3%)	155 (31.1%)	58 (24.8%)
Easily	203 (8.6%)	49 (7.2%)	117 (22.5%)	13 (3.1%)	17 (3.4%)	7 (3.0%)
Problems with daily living tasks due to physical or mental impairment						
Yes	471 (20.0%)	102 (14.9%)	166 (32.0%)	60 (14.3%)	109 (21.8%)	34 (14.5%)
No	1886 (80.0%)	583 (85.1%)	353 (68.0%)	360 (85.7%)	390 (78.2%)	200 (85.5%)
BMI ≥30 kg/m^2^						
Yes	403 (17.1%)	102 (14.9%)	85 (16.4%)	76 (18.1%)	90 (18.0%)	50 (21.4%)
No	1954 (82.9%)	583 (85.1%)	434 (83.6%)	344 (81.9%)	409 (82.0%)	184 (78.6%)
Pneumopathy						
Yes	516 (21.9%)	136 (19.9%)	96 (18.5%)	95 (22.6%)	127 (25.5%)	62 (26.5%)
No	1841 (78.1%)	549 (80.1%)	423 (81.5%)	325 (77.4%)	372 (74.5%)	172 (73.5%)
Cardiopathy						
Yes	575 (24.4%)	158 (23.1%)	123 (23.7%)	96 (22.9%)	144 (28.9%)	54 (23.1%)
No	1782 (75.6%)	527 (76.9%)	396 (76.3%)	324 (77.1%)	355 (71.1%)	180 (76.9%)
Diabetes						
Yes	750 (31.8%)	177 (25.8%)	231 (44.5%)	105 (25.0%)	160 (32.1%)	77 (32.9%)
No	1607 (68.2%)	508 (74.2%)	288 (55.5%)	315 (75.0%)	339 (67.9%)	157 (67.1%)

NUTS Nomenclature of Territorial Units for Statistics, BMI body mass index Northwestern Italy includes the regions of Piedmont, Aosta Valley, Lombardy, and Liguria; Northeastern Italy includes the regions of Trentino-South Tyrol, Veneto, Friuli-Venezia Giulia, and Emilia-Romagna; Central Italy includes the regions of Tuscany, Umbria, Marche, and Lazio; Southern Italy includes the regions of Abruzzo, Molise, Campania, Apulia, Basilicata, and Calabria; Insular Italy includes the regions of Sicily and Sardinia. BMI, body mass index

*According to the Eurostat Degree of Urbanization (DEGURBA) classification system

### Children

**Fig. 8:**
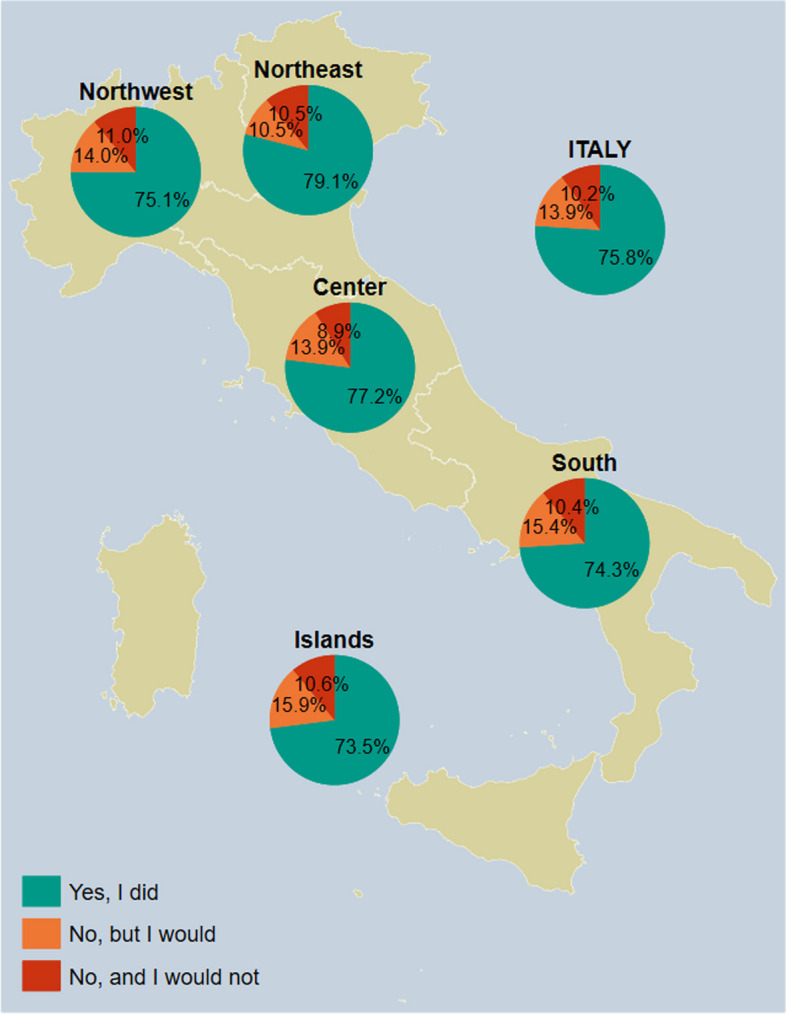
Pneumococcal vaccine uptake among respondents who answered on their children’s behalf (*n* = 1064), overall and by NUTS statistical region; if the answer is no, the respondents are asked whether or not they would get their children vaccinated. *Notes:* Northwestern Italy includes the regions of Piedmont, Aosta Valley, Lombardy, and Liguria; Northeastern Italy includes the regions of Trentino-South Tyrol, Veneto, Friuli-Venezia Giulia, and Emilia-Romagna; Central Italy includes the regions of Tuscany, Umbria, Marche, and Lazio; Southern Italy includes the regions of Abruzzo, Molise, Campania, Apulia, Basilicata, and Calabria; Insular Italy includes the regions of Sicily and Sardinia. *NUTS*, Nomenclature of Territorial Units for Statistics

**Fig. 9:**
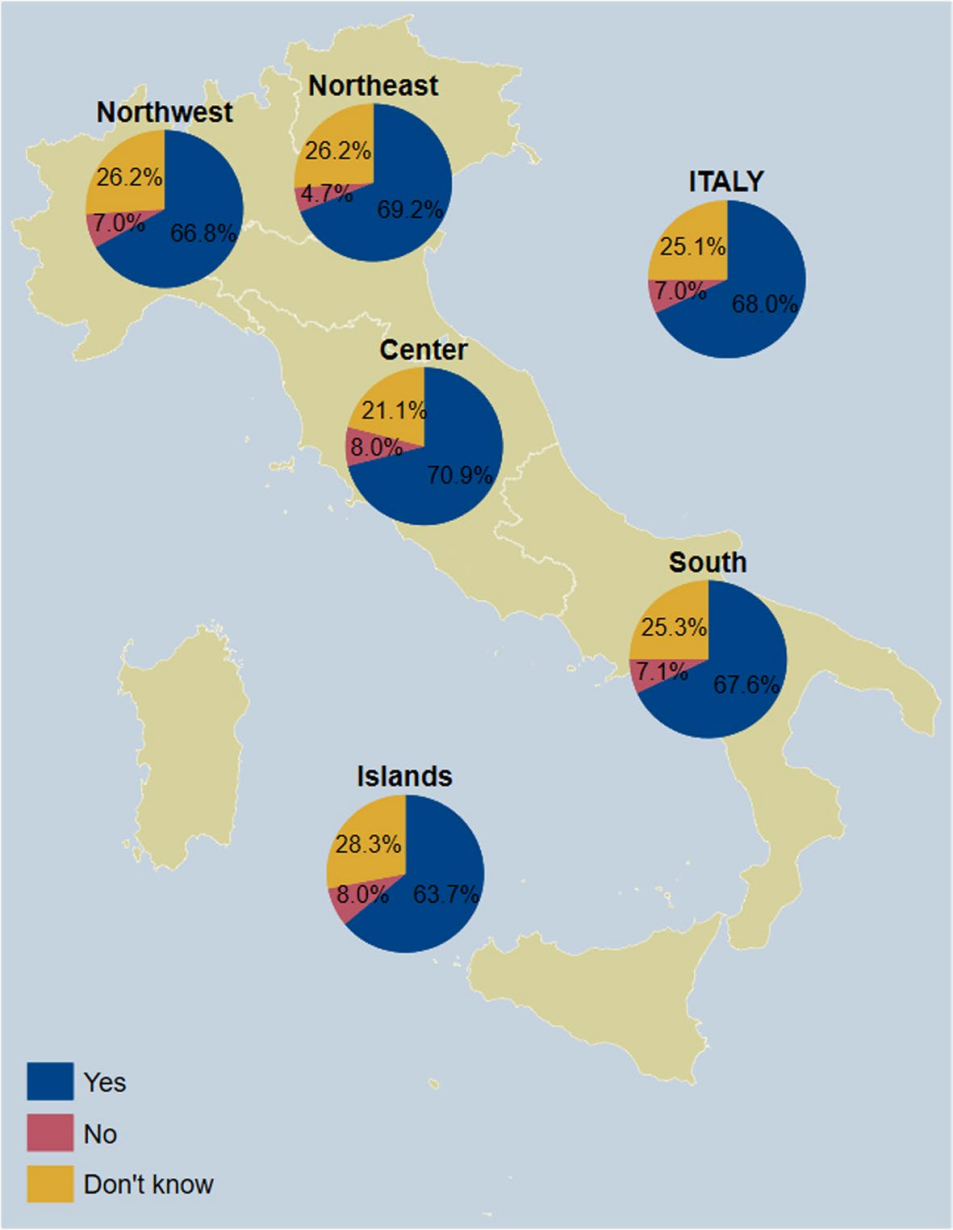
Awareness that children have higher priority for pneumococcal vaccination (*n* = 1064), overall and by NUTS statistical region. *Notes:* Northwestern Italy includes the regions of Piedmont, Aosta Valley, Lombardy, and Liguria; Northeastern Italy includes the regions of Trentino-South Tyrol, Veneto, Friuli-Venezia Giulia, and Emilia-Romagna; Central Italy includes the regions of Tuscany, Umbria, Marche, and Lazio; Southern Italy includes the regions of Abruzzo, Molise, Campania, Apulia, Basilicata, and Calabria; Insular Italy includes the regions of Sicily and Sardinia. *NUTS*, Nomenclature of Territorial Units for Statistics

Among the 1064 respondents who answered on their youngest child’s behalf, 640 (60.2%) were women and mean age was 38.2 ± 7.3 (Table 2). The child’s gender was reported to be female by 500 respondents (47.0%), while mean age was 3.9 ± 2.5. Overall, 807 children (75.8%) had received a pneumococcal vaccine, 148 (13.9%) had not but their parents wanted to, and 100 (10.2%) had not and their parents did not want to. The highest uptake was registered in the Northeast, at 79.1%, while the lowest was registered in the Islands, at 73.5% (Fig. 8). Overall, 723 respondents (68.0%) believed their child had the right to pneumococcal vaccination free of charge, while 74 (7.0%) believed they had not and 267 (25.1%) did not know. The highest proportion of respondents that believed their child had the right to free vaccination was registered in Central Italy, at 70.9%, while the lowest proportion was registered in the Islands, at 63.7% (Fig. 9). As for disease risk perception, 128 parents (12.0%) were very worried for their child about pneumococcal disease, 376 (35.3%) quite worried, 436 (41.0%) a little worried, and 124 (11.7%) not worried. The highest proportion of parents who were very or quite worried was registered in the Islands, at 53.1%, while the lowest proportion was registered in the Northeast, at 41.8%. Overall, 255 parents (24.0%) viewed the vaccine as very safe, 685 (64.5%) as quite safe, 95 (8.9%) as quite unsafe, and 29 (2.7%) as very unsafe. The highest proportion of parents who thought that the vaccine was quite or very safe was registered in Central Italy, at 90.7%, while the lowest rate was registered in the Northeast, at 81.4%. With regard to perceived ease of access to vaccination, 248 parents (23.3%) found it very easy to access vaccination for their child, 668 (62.8%) quite easy, 119 (11.2%) quite difficult, and 29 (2.7%) very difficult. The highest proportion of parents who viewed access to vaccination for their child as very or quite easy was registered in Central Italy, at 89.5%, while the lowest proportion was registered in the Islands, at 71.4%.

**Table 2:** Sociodemographic characteristics of the study respondents who provided information about their youngest children’s pneumococcal vaccine uptake, overall and by NUTS statistical region

Characteristic	Italy	Northwestern Italy	Northeastern Italy	Central Italy	Southern Italy	Insular Italy
	(*n* = 1064)	(*n* = 301)	(*n* = 172)	(*n* = 237)	(*n* = 241)	(*n* = 113)
Gender						
Male	423 (39.8%)	123 (40.9%)	56 (32.6%)	104 (43.9%)	103 (42.7%)	37 (32.7%)
Female	640 (60.2%)	178 (59.1%)	116 (67.4%)	133 (56.1%)	138 (57.3%)	75 (66.4%)
Non-binary	1 (0.1%)	0 (0.0%)	0 (0.0%)	0 (0.0%)	0 (0.0%)	1 (0.9%)
Age group, y						
18–24	32 (3.0%)	7 (2.3%)	7 (4.1%)	4 (1.7%)	8 (3.3%)	6 (5.3%)
25–34	260 (24.4%)	69 (22.9%)	48 (27.9%)	59 (24.9%)	63 (26.1%)	21 (18.6%)
35–44	551 (51.8%)	151 (50.2%)	84 (48.8%)	132 (55.7%)	115 (47.7%)	69 (61.1%)
45–54	198 (18.6%)	66 (21.9%)	29 (16.9%)	39 (16.5%)	48 (19.9%)	16 (14.2%)
55–64	23 (2.2%)	8 (2.7%)	4 (2.3%)	3 (1.3%)	7 (2.9%)	1 (0.9%)
Place of residence degree of urbanization*						
City (densely populated area)	417 (39.2%)	133 (44.2%)	48 (27.9%)	106 (44.7%)	100 (41.5%)	30 (26.5%)
Town or suburb (intermediate density area)	485 (45.6%)	136 (45.2%)	87 (50.6%)	97 (40.9%)	103 (42.7%)	62 (54.9%)
Rural area (thinly populated area)	162 (15.2%)	32 (10.6%)	37 (21.5%)	34 (14.3%)	38 (15.8%)	21 (18.6%)
Educational attainment						
Less than high school diploma	91 (8.6%)	28 (9.3%)	17 (9.9%)	26 (11.0%)	13 (5.4%)	7 (6.2%)
High school diploma	593 (55.7%)	181 (60.1%)	91 (52.9%)	128 (54.0%)	122 (50.6%)	71 (62.8%)
Academic degree	272 (25.6%)	62 (20.6%)	46 (26.7%)	62 (26.2%)	77 (32.0%)	25 (22.1%)
Post-graduate/Doctorate degree	108 (10.2%)	30 (10.0%)	18 (10.5%)	21 (8.9%)	29 (12.0%)	10 (8.8%)
Household composition						
Alone	28 (2.6%)	10 (3.3%)	4 (2.3%)	6 (2.5%)	7 (2.9%)	1 (0.9%)
Couple	875 (82.2%)	251 (83.4%)	137 (79.7%)	195 (82.3%)	193 (80.1%)	99 (87.6%)
With parents/family	85 (8.0%)	15 (5.0%)	19 (11.0%)	21 (8.9%)	23 (9.5%)	7 (6.2%)
Other	76 (7.1%)	25 (8.3%)	12 (7.0%)	15 (6.3%)	18 (7.5%)	6 (5.3%)
Able to pay for things needed in life						
With great difficulty	102 (9.6%)	24 (8.0%)	16 (9.3%)	23 (9.7%)	22 (9.1%)	17 (15.0%)
With some difficulty	502 (47.2%)	131 (43.5%)	93 (54.1%)	107 (45.1%)	113 (46.9%)	58 (51.3%)
Quite easily	422 (39.7%)	131 (43.5%)	52 (30.2%)	102 (43.0%)	101 (41.9%)	36 (31.9%)
Easily	38 (3.6%)	15 (5.0%)	11 (6.4%)	5 (2.1%)	5 (2.1%)	2 (1.8%)

NUTS Nomenclature of Territorial Units for Statistics Northwestern Italy includes the regions of Piedmont, Aosta Valley, Lombardy, and Liguria; Northeastern Italy includes the regions of Trentino-South Tyrol, Veneto, Friuli-Venezia Giulia, and Emilia-Romagna; Central Italy includes the regions of Tuscany, Umbria, Marche, and Lazio; Southern Italy includes the regions of Abruzzo, Molise, Campania, Apulia, Basilicata, and Calabria; Insular Italy includes the regions of Sicily and Sardinia.

*According to the Eurostat Degree of Urbanization (DEGURBA) classification system

### Multivariable analysis

As shown in Table 3, older age and lack of awareness of having higher priority for pneumococcal vaccination were significantly associated with not being vaccinated. Moreover, identifying as female, living in rural areas, having less than high school education, not being worried about pneumococcal pneumonia, perceiving pneumococcal vaccines as unsafe, and having friends or relatives against vaccination were significantly associated with not having been vaccinated and not wanting to, while self-reported difficulty in access to healthcare was a significant predictor of not having been vaccinated but wanting to. We also found that suffering from pneumopathy, heart disease and diabetes significantly increased the probability of vaccine uptake, as well as living in Northeastern or Central Italy. The analysis of possible interaction effects across covariates revealed that the impact of dear ones’ opinions on vaccine refusal was much stronger in rural areas (unfavorable/very unfavorable: 44.9%; very favorable: 12.7%; Δ = + 32.3; 95% CI = 16.6 to 48.0) than in towns/suburbs (unfavorable/very unfavorable: 20.9%; very favorable: 26.5%; Δ = + 5.6; 95% CI = − 2.8 to 14.1) and cities (unfavorable/very unfavorable: 26.6%; very favorable: 17.2%; Δ = + 9.4; 95% CI = 0.3 to 18.5) (LR test = 33.3, *p*-value = 0.0067). With regard to vaccine uptake among children (Table 4), a significant predictor of not having been vaccinated was parents not being aware their child had higher priority for pneumococcal vaccination. Safety concerns were significantly associated with higher probability of not wanting their child to get vaccinated, while self-reported difficulties in access to healthcare were significantly associated with higher probability of not being but wanting to be vaccinated. †Including non-binary people

**Table 3:** Results of multivariable multinomial logistic regression analysis: determinants of pneumococcal vaccine uptake and hesitancy (expressed as delay vs. refusal) among respondents who answered on their own behalf (*n* = 2357)

Characteristic	Did get the vaccine			Would get the vaccine			Would not get the vaccine		
	Predicted	Discrete difference (*Δ*)		Predicted	Discrete difference (*Δ*)		Predicted	Discrete difference (*Δ*)	
	probability	Estimate	95% CI	probability	Estimate	95% CI	probability	Estimate	95% CI
Gender									
Male	40.6%	Ref.		34.4%	Ref.		25.0%	Ref.	
Female†	38.0%	−2.7	−5.8, 0.4	34.1%	−0.3	−3.9, 3.2	28.0%	3.0*	0.3, 5.8
Age group, y									
18–34	48.5%	Ref.		27.8%	Ref.		23.7%	Ref.	
35–44	51.3%	2.8	−4.0, 9.6	25.9%	−1.9	−9.2, 5.5	22.8%	−0.9	−7.3, 5.5
45–54	35.9%	−12.6*	−19.4, −5.8	31.6%	3.8	−3.8, 11.4	32.5%	8.8*	2.4, 15.2
55–64	30.4%	−18.1*	−24.9, − 11.3	39.5%	11.7*	4.0, 19.4	30.0%	6.4	0.0, 12.8
≥ 65	37.6%	−10.9*	− 16.9, − 4.9	36.7%	8.9*	2.2, 15.6	25.7%	2.0	−3.7, 7.8
NUTS statistical region									
Northwestern Italy	34.9%	Ref.		37.4%	Ref.		27.7%	Ref.	
Northeastern Italy	48.6%	13.7*	9.0, 18.4	26.8%	−10.7*	−15.9, −5.5	24.6%	−3.1	−7.2, 1.1
Central Italy	42.7%	7.8*	3.2, 12.4	30.7%	−6.7*	−11.8, −1.6	26.6%	−1.1	−5.0, 2.8
Southern Italy	37.6%	2.7	−1.7, 7.1	37.8%	0.4	−4.6, 5.4	24.6%	−3.1	−6.9, 0.6
Insular Italy	32.7%	−2.2	−7.8, 3.4	38.0%	0.6	−5.9, 7.1	29.3%	1.6	−3.4, 6.6
Degree of urbanization‡									
City	39.3%	Ref.		35.4%	Ref.		25.2%	Ref.	
Town or suburb	39.7%	0.4	−3.0, 3.7	34.1%	−1.3	−5.0, 2.5	26.2%	0.9	−2.0, 3.9
Rural area	39.1%	−0.3	−5.2, 4.6	30.5%	−4.9	−10.5, 0.7	30.4%	5.2*	0.7, 9.7
Educational attainment									
Post-graduate/Doctorate degree	45.2%	Ref.		31.5%	Ref.		23.3%	Ref.	
Academic degree	40.1%	−5.1	−11.2, 1.1	35.9%	4.4	−2.6, 11.5	23.9%	0.6	−5.2, 6.5
High school diploma	38.9%	−6.3*	−12.0, −0.6	34.3%	2.9	−3.6, 9.3	26.7%	3.4	−1.9, 8.8
Less than high school diploma	36.6%	−8.6*	−15.3, − 1.8	34.1%	2.6	−5.0, 10.3	29.2%	5.9*	0.0, 11.9
Pneumopathy									
No	38.0%	Ref.		33.7%	Ref.		28.3%	Ref.	
Yes	44.2%	6.2*	1.9, 10.6	36.6%	2.9	−2.2, 8.0	19.2%	−9.1*	−13.0, −5.2
Cardiopathy									
No	38.0%	Ref.		33.6%	Ref.		28.4%	Ref.	
Yes	44.0%	6.0*	2.1, 9.9	36.4%	2.8	−1.7, 7.3	19.6%	−8.8*	−12.2, −5.3
Diabetes									
No	37.5%	Ref.		34.4%	Ref.		28.1%	Ref.	
Yes	44.2%	6.8*	2.9, 10.7	34.2%	−0.3	−4.7, 4.2	21.6%	−6.5*	−10.0, −3.1
Worry about pneumococcal pneumonia									
Very worried	51.0%	Ref.		32.3%	Ref.		16.7%	Ref.	
Quite worried	41.5%	−9.5*	−17.5, −1.5	45.0%	12.7*	3.2, 22.1	13.6%	−3.2	−12.2, 5.9
A little worried	38.4%	−12.6*	−20.5, −4.7	35.6%	3.3	−6.0, 12.5	26.0%	9.3*	0.4, 18.3
Not worried	36.6%	−14.4*	−22.7, −6.2	21.0%	−11.3*	−20.9, −1.7	42.5%	25.8*	16.3, 35.2
Perception of vaccine safety									
Very safe	46.9%	Ref.		40.5%	Ref.		12.6%	Ref.	
Quite safe	39.0%	−7.9*	−12.5, −3.3	37.0%	−3.5	−8.8, 1.7	24.0%	11.4*	7.5, 15.4
Quite/Very unsafe	29.7%	−17.3*	−24.1, − 10.4	17.0%	−23.5*	−30.5, −16.4	53.3%	40.7*	34.1, 47.3
Dear ones’ views on vaccination in general									
Very favorable	41.0%	Ref.		40.4%	Ref.		18.5%	Ref.	
Favorable	41.5%	0.4	−3.9, 4.7	35.3%	−5.2*	−10.1, −0.3	23.3%	4.8*	1.0, 8.5
Quite favorable	37.1%	−4.0	−8.5, 0.6	32.1%	−8.4*	−13.5, − 3.3	30.8%	12.3*	8.3, 16.4
Quite unfavorable	36.0%	−5.1	−11.8, 1.7	25.8%	−14.7*	−22.2, −7.1	38.2%	19.7*	13.6, 25.9
Unfavorable/Very unfavorable	42.1%	1.1	−6.3, 8.4	28.5%	−12.0*	−20.2, −3.7	29.4%	10.9*	4.8, 17.0
Awareness of having priority for vaccination									
Yes	59.3%	Ref.		27.6%	Ref.		13.1%	Ref.	
No	18.7%	−40.5*	−45.7, −35.4	39.0%	11.4*	5.4, 17.3	42.3%	29.2*	24.1, 34.2
Don’t know	21.5%	−37.8*	−41.9, − 33.6	46.3%	18.6*	14.3, 23.0	32.2%	19.1*	15.8, 22.4
Perceived ease of access to get the vaccine									
Very easy	45.3%	Ref.		27.1%	Ref.		27.5%	Ref.	
Quite easy	37.9%	−7.4*	−11.9, −2.9	34.2%	7.1*	1.9, 12.2	27.9%	0.4	−4.3, 5.0
Quite/Very difficult	37.1%	−8.2*	−13.9, −2.6	40.5%	13.4*	7.0, 19.7	22.4%	−5.1*	−10.2, 0.0

NUTS, Nomenclature of Territorial Units for Statistics

†Including non-binary people

‡According to the Eurostat Degree of Urbanization (DEGURBA) classification system

*P-value ≤0.05, that is, Δ significantly ≠0

**Table 4:** Results of multivariable multinomial logistic regression analysis: determinants of pneumococcal vaccine uptake and hesitancy (expressed as delay vs. refusal) among respondents who answered on their children’s behalf (*n* = 1064)

Characteristic	Did get the vaccine			Would get the vaccine			Would not get the vaccine		
	Predicted	Discrete difference (*Δ*)		Predicted	Discrete difference (*Δ*)		Predicted	Discrete difference (*Δ*)	
	probability	Estimate	95% CI	probability	Estimate	95% CI	probability	Estimate	95% CI
Parent’s gender									
Male	74.5%	Ref.		16.1%	Ref.		9.4%	Ref.	
Female†	76.9%	2.4	−2.2, 7.1	12.1%	−4.0	−8.3, 0.2	11.0%	1.6	−1.3, 4.6
Parent’s age group, y									
18–34	76.4%	Ref.		14.6%	Ref.		9.0%	Ref.	
35–44	77.2%	0.8	−4.6, 6.2	13.4%	−1.2	−6.0, 3.7	9.4%	0.4	−3.2, 4.0
45–64	72.0%	−4.4	−11.2, 2.3	14.4%	−0.2	−6.2, 5.8	13.6%	4.7	−0.1, 9.4
NUTS statistical region									
Northwestern Italy	74.9%	Ref.		13.9%	Ref.		11.3%	Ref.	
Northeastern Italy	80.2%	5.3	−1.3, 12.0	11.8%	−2.1	−8.1, 4.0	8.0%	−3.2	−7.6, 1.1
Central Italy	74.8%	−0.1	−6.4, 6.2	14.8%	1.0	−4.7, 6.7	10.4%	−0.9	−5.2, 3.4
Southern Italy	75.6%	0.7	−5.4, 6.8	14.1%	0.2	−5.3, 5.7	10.3%	−0.9	−5.1, 3.3
Insular Italy	74.0%	−0.9	−8.7, 6.9	14.7%	0.9	−6.2, 7.9	11.3%	0.0	−5.3, 5.3
Degree of urbanization‡									
City	74.3%	Ref.		14.3%	Ref.		11.4%	Ref.	
Town or suburb	75.7%	1.4	−3.4, 6.2	14.7%	0.4	−4.0, 4.8	9.5%	−1.8	−5.1, 1.4
Rural area	79.9%	5.6	−0.7, 11.9	10.3%	−4.0	−9.6, 1.5	9.8%	−1.5	−5.9, 2.8
Parent’s educational attainment									
Post-graduate/Doctorate degree	74.8%	Ref.		16.0%	Ref.		9.2%	Ref.	
Academic degree	69.6%	−5.2	−13.4, 3.0	17.1%	1.1	−6.4, 8.6	13.4%	4.1	−1.4, 9.6
High school diploma	77.7%	2.9	−4.6, 10.5	12.8%	−3.2	−10.2, 3.8	9.5%	0.3	− 4.5, 5.1
Less than high school diploma	83.8%	9.1	−0.4, 18.5	7.9%	−8.1	−16.5, 0.3	8.3%	−1.0	−7.4, 5.5
Child’s gender									
Male	75.1%	Ref.		14.3%	Ref.		10.6%	Ref.	
Female	76.7%	1.6	−2.8, 6.0	13.4%	−0.9	−4.9, 3.1	9.9%	−0.7	−3.7, 2.2
Child’s age group									
9 wk. to 3 y	76.7%	Ref.		13.8%	Ref.		9.5%	Ref.	
4 to 5 y	74.7%	−2.0	−7.5, 3.5	13.7%	−0.2	−5.1, 4.8	11.6%	2.2	−1.6, 5.9
6 to 8 y	75.5%	−1.1	−6.4, 4.2	14.3%	0.5	−4.4, 5.3	10.1%	0.7	−2.8, 4.2
Worry about pneumococcal pneumonia									
Very worried	79.7%	Ref.		11.9%	Ref.		8.4%	Ref.	
Quite worried	77.0%	−2.7	−10.7, 5.2	16.7%	4.8	−2.4, 12.0	6.3%	−2.1	−7.9, 3.8
A little worried	73.2%	−6.6	−14.4, 1.3	14.3%	2.4	−4.6, 9.5	12.5%	4.1	−1.8, 10.0
Not worried	80.9%	1.2	−7.8, 10.1	6.9%	−5.0	−12.6, 2.6	12.2%	3.9	−3.2, 10.9
Perception of vaccine safety for the child									
Very safe	87.2%	Ref.		11.5%	Ref.		1.2%	Ref.	
Quite safe	77.5%	−9.7*	−15.2, −4.1	16.0%	4.5	−0.8, 9.8	6.4%	5.2*	2.6, 7.8
Quite/Very unsafe	52.0%	−35.2*	−46.6, −23.9	12.2%	0.7	−7.5, 8.9	35.8%	34.5*	24.6, 44.4
Dear ones’ views on vaccination in general									
Very favorable	74.4%	Ref.		17.5%	Ref.		8.1%	Ref.	
Favorable	74.6%	0.2	−6.6, 6.9	16.4%	−1.1	−7.5, 5.3	9.0%	0.9	−4.1, 5.9
Quite favorable	79.3%	4.8	−1.7, 11.4	10.2%	−7.3*	−13.4, − 1.3	10.6%	2.5	−2.4, 7.4
Quite unfavorable	73.6%	−0.9	−10.8, 9.1	16.4%	−1.1	−10.5, 8.4	10.0%	1.9	−4.2, 8.1
Unfavorable/Very unfavorable	75.2%	0.7	−10.8, 12.3	10.2%	−7.3	−17.5, 2.9	14.6%	6.6	−1.3, 14.4
Awareness that the child has priority for vaccination									
Yes	86.3%	Ref.		8.6%	Ref.		5.1%	Ref.	
No	53.4%	−32.9*	−45.0, −20.9	25.9%	17.3*	6.4, 28.2	20.7%	15.6*	7.7, 23.5
Don’t know	57.6%	−28.7*	−34.9, −22.5	26.1%	17.5*	11.9, 23.2	16.3%	11.2*	7.2, 15.2
Perceived ease of access for the child to get the vaccine									
Very easy	79.7%	Ref.		11.0%	Ref.		9.3%	Ref.	
Quite easy	76.2%	−3.5	−9.7, 2.7	12.8%	1.8	−3.7, 7.3	11.0%	1.7	−2.9, 6.4
Quite/Very difficult	68.8%	−10.9*	−19.9, −1.9	22.1%	11.1*	2.5, 19.6	9.1%	−0.2	−5.4, 5.0

NUTS, Nomenclature of Territorial Units for Statistics

†Including non-binary people

‡According to the Eurostat Degree of Urbanization (DEGURBA) classification system

*P-value ≤0.05, that is, *Δ* significantly ≠ 0

## Discussion

In the context of this extensive cross-sectional study conducted in Italy, it was observed that approximately one third of the total respondents had undergone pneumococcal vaccination in various forms. This finding is particularly remarkable, as it pertains not to the general population but specifically to participants who align with the criteria established by the national vaccination campaign objectives, and could therefore have been vaccinated free of charge. Another notable finding concerns the attitude of respondents who have not received the vaccination. The majority are willing to get vaccinated, indicating that their attitude towards vaccination cannot be labeled as vaccine hesitancy, according to the most recent definition by the SAGE group [17]. Moreover, a perceived difficulty in accessing vaccination facilities emerged as a significant predictor of this attitude, rather than simply not wanting to get vaccinated. Combined with the fact that nearly half of the respondents were unaware of having free access to vaccination and exhibited an overall high level of trust in vaccine safety, this observation suggests that lack of information about pneumococcal vaccination and improving access to healthcare services are critical targets for enhancing uptake in Italy. We observed gender differences in vaccination uptake, which were confirmed through multivariable analysis. Respondents identifying as female were found to be less likely to be vaccinated and less willing to receive the vaccine. Interestingly, these differences did not appear to be linked to underlying concerns about vaccine safety or perceptions of disease risk, as attitudes seemed similar across genders. On the other hand, female respondents seemed to face more challenges in accessing vaccination services. This could be attributed to their more frequent interactions with the Italian National Health Service, considering the disproportionate burden on women in Italian society to take care of family members’ health. It is noteworthy that women still contribute to 65.7% of the time spent by Italian households on caring for children and/or other family members [20]. In our study, uptake figures present relevant territorial differences. These can be at least partially explained by the different strategies adopted in different regions when it comes to enacting pneumococcal vaccination campaigns: according to a report by the Italian Consortium for Applied Economic Research in Healthcare (*CREA Sanità*), the only regions that have region-wide agreements with professional associations that compel general practitioners to take part in the campaign are Emilia-Romagna and Friuli-Venezia Giulia, whereas other regions make it optional or have only local agreements. Moreover, according to the same report, the regions that have an active call system to recruit target populations are Emilia-Romagna, Veneto, the autonomous province of Trento, Abruzzo, Lombardy, and Liguria [21]. This matches our finding of a disproportionately high uptake in Northeastern Italy, which was also confirmed by multivariable analysis: regions in this area (Emilia-Romagna, Veneto, and Friuli-Venezia Giulia, autonomous regions of Trento and Bolzano) appear to have enacted more structured and pervasive vaccination strategies. Our survey found uptake rates for pneumococcal vaccination to be higher than those from recent surveys and other cross-sectional studies in European countries among older adults (9.6–39.0%) and other at-risk groups: individuals with diabetes mellitus (12.9–23.7%), chronic heart disease (11.6–18.7%) and chronic respiratory disease (13.9–44.4%) [22–26]. Moreover, our results appear higher than those derived from the scarce literature on coverage in Italy among older adults (15.1–26.3%); however, it must be noted that data collection in available studies dates back to 2004 and 2017, and advancements in coverage rates are plausible since then [27, 28]. When comparing our results with other available studies, it should also be noted that we have chosen to exclude from calculations respondents who were not sure about their vaccination status, in order to increase reliability of further answers. Our uptake data in children diverges from the available official figures on vaccination coverage in this demographic. Official coverage data from vaccination registries sets coverage among children at over 90% among the cohorts for which vaccination was made freely available and actively offered from 2017 on [29]. It is worth mentioning that our uptake figure refers to children aged 9 weeks to 9 years, including cohorts that have not been actively offered vaccination, which might partially explain such discrepancy [16]. Moreover, a significant recall bias should be expected when inquiring about children’s vaccination from parents, as childhood vaccinations are often many and administered in a relatively narrow timeframe. Among parents answering on behalf of their children, not knowing their child has a right to free vaccination and perceived difficulty in accessing vaccination facilities are confirmed as negative predictors of uptake, mirroring the results found among adults answering for themselves. Pneumococcal vaccination coverage rates among at-risk adults appear unsatisfactory and those among children still have room for improvement. As studied for other vaccinations in the Italian context [30], the need for effective vaccination campaigns employing age-appropriate methods is justified not only by the direct individual benefit of pneumococcal vaccination, but also by the notion that a large component of the effect on IPD incidence in at-risk adults has been explained through herd immunity and reduction of asymptomatic carriage by healthy individuals [31]. A secondary but not less important benefit comes from the well documented decline in antibiotic resistant serotypes after the introduction of PCV-7 vaccination in children in Europe and the USA [32]. Relationship between immunization and antibiotic resistance is complex, as is shown by the subsequent increasing trend in resistant non-PCV-7 serotypes that was only interrupted by the introduction of PCV-13 vaccination. Nonetheless, a relevant albeit temporary effect on resistance must be recognized as an ulterior means of resistance control, in a situation where new antimicrobial drugs struggle to keep up with the appearance of new forms of resistance. Recent introduction of immunization against an even wider range of serotypes with PCV-15 and PCV-20, is likely to see a repetition in this pattern.

Our study presents some limitations that should be taken into account when interpreting the results. As with all cross-sectional studies, some degree of recall bias is to be expected. To reduce the effect of this bias, we have directed questions to parents regarding their children’s vaccination status only towards their youngest child, for which administration times are likely to be closer in time to the data collection timeframe. Additionally, the CAWI interviewing methodology has the intrinsic potential to introduce some degree of selection bias in the sample, usually skewing it towards younger, more educated and “tech-savvy” individuals. Moreover, we decided to exclude respondents who were unsure of their vaccination status from the remaining questions in the questionnaire section, to make the answers more reliable. This may have introduced some degree of selection bias in our analyses, as it may not be safe to assume that respondents who do not remember their vaccination status are equally likely to be vaccinated or unvaccinated. Another factor to take into account when interpreting our results is that the list of conditions we used to grant access to the pneumococcal vaccination section is not exhaustive. We made the choice to target respondents with the most prevalent conditions in order to contain the length and complexity of the questionnaire, and to increase answer reliability. Our results should therefore be interpreted while keeping in mind that the study sample represents individuals with the most prevalent and easily assessed conditions, and not the entire population that has the right to free pneumococcal vaccination in Italy.

## Conclusion

At the moment, our study represents one of the very few sources of information regarding pneumococcal vaccine uptake among Italian citizens and at-risk categories. Uptake levels appear inadequate both among high-risk adults and children. Information on vaccine availability and accessibility of vaccination services emerge as areas for priority intervention in order to increase uptake. Specifically, when it comes to adults with comorbidities, a larger effort should be made in in involving mainly GPs, but also other health professionals that interact with the patients throughout the Health System in conducting full vaccination schedule assessments and referral to vaccination services when needed. HCW are still one of the most relevant actors in helping patients navigate health services and in gaining their trust: they should be provided with adequate assistance in the form of organization and technology to allow for a more systematic and standardized vaccine anamnesis and prompt referral to vaccination services. The often-large territorial inequities in vaccine coverage that result from a lack of homogeneity in vaccinal practices among HCW, and therefore ignorance about vaccine rights, are unacceptable. In this respect, our work might represent a useful tool to inform political decision-making in an ever-changing environment. Indeed, the recent introduction of two conjugate vaccines, the changes in serotype epidemiology, and antimicrobial resistance make it crucial to have real-time and readily available information on vaccination uptake.

### Supplementary Information

**Supplementary Material 1.**

**Supplementary Material 2.**

**Supplementary Material 3.**

**Supplementary Material 4.**

**Supplementary Material 5.**

**Supplementary Material 6.**

## Data Availability

The datasets on which this study in based can be made available on request by contacting the corresponding author.
