# Supplementary material for: Pneumococcal vaccine uptake among high-risk adults and children in Italy: results from the OBVIOUS project survey
Source: BMC Public Health. 2024 Mar 7;24:736. doi: 10.1186/s12889-024-18216-3 (PMC10921627; doi:10.1186/s12889-024-18216-3)
Supplement: Supplementary file 1 — Supplementary Material 1. [file 12889_2024_18216_MOESM1_ESM.docx]

## Questionnaire. English translation of the questionnaire used for the survey.

1. What is your gender?
   1. Female
   2. Male
   3. Non-Binary
2. Birth date
3. What is your education level?
   1. Elementary/middle school
   2. High school
   3. University
   4. Postgraduate education
4. What is the postcode of the area you live in?
5. What is your current occupation?
   1. Student
   2. Doctor
   3. Other health worker
   4. Law enforcement
   5. Teacher
   6. Employed (other category than above)
   7. Unemployed
   8. Retired
6. Who do you live with?
   1. I live alone
   2. I live as a couple
   3. I live with my family of origin
   4. Other
7. With the financial resources available to you (from your own or your family's income) are you able to meet the needs of your current living situation?
   1. grade 1 (with many difficulties)
   2. grade 2
   3. grade 3
   4. grade 4
   5. grade 5 (with no difficulties)
8. Do you have any children?
   1. Yes
   2. No
9. Is your youngest child…?
   1. Male
   2. Female
10. What is your youngest child’s date of birth?
11. Who is the main decision-maker when it comes to vaccination of your children?
    1. Mainly me
    2. Mainly my partner
    3. Evenly divided
12. Were you pregnant at the beginning of the influenza epidemic season (October/November 2021)
    1. Yes
    2. No
13. Do you have difficulties completing daily tasks such as going to the doctor or buying groceries, due to a physical/psychological/sensory disability?
    1. Yes
    2. No
14. What is your weight?
15. What is your height?
16. Do you have a chronic respiratory condition (i.e.: severe asthma, bronchopulmonary dysplasia, cystic fibrosis and chronic obstructive pulmonary disease-BPCO)?
    1. Yes
    2. No
17. Do you have a chronic cardiovascular condition (i.e.: congenital and acquired heart disease)?
    1. Yes
    2. No
18. Do you have diabetes?
    1. Yes
    2. No
19. In which of the following facilities did you have most of your vaccinations?
    1. Hospital
    2. Pharmacy
    3. At your General Practitioner’s
    4. Vaccination hub
    5. At home
    6. At work
20. If you could choose, in which facility/place would you prefer to receive a vaccination?
    1. Hospital
    2. Pharmacy
    3. At your General Practitioner’s
    4. Vaccination hub
    5. At home
    6. At work
21. How do your close family and friends feel about vaccines?
    1. grade 1 (very unfavorable)
    2. grade 2
    3. grade 3
    4. grade 4
    5. grade 5 (very favorable)
22. Did you ever receive/Has your youngest child ever received a pneumococcal vaccination?
    1. Yes
    2. No
    3. I’m not sure
23. Would you get vaccinated/let your youngest child get vaccinated with a pneumococcal vaccine?
    1. Yes
    2. No
    3. I’m not sure
24. How worried are you about you/your youngest child getting sick with pneumococcal pneumonia?
    1. grade 1 (not worried)
    2. grade 2 (a little worried)
    3. grade 3 (quite worried)
    4. grade 4 (very worried)
25. How safe do you think pneumococcal vaccines are/for your youngest child?
    1. grade 1 (very unsafe)
    2. grade 2 (quite unsafe)
    3. grade 3 (quite safe)
    4. grade 4 (very safe)
26. Do you think you/your youngest child currently has the right to free pneumococcal vaccination?
    1. Yes
    2. No
    3. I don’t know
27. How easy do you think it would be to get pneumococcal vaccination/for your youngest child?
    1. grade 1 (very hard)
    2. grade 2 (quite hard)
    3. grade 3 (quite easy)
    4. grade 4 (very easy)
