# Supplementary material for: Pneumococcal vaccine uptake among high-risk adults and children in Italy: results from the OBVIOUS project survey
Source: BMC Public Health. 2024 Mar 7;24:736. doi: 10.1186/s12889-024-18216-3 (PMC10921627; doi:10.1186/s12889-024-18216-3)
Supplement: Supplementary file 2 — Supplementary Material 2. [file 12889_2024_18216_MOESM2_ESM.docx]

## Additional Table 1. Uptake, awareness, and ease of access among respondents who answered on their own behalf (*n* = 2357), by region.

| Characteristic | PIE | VDA | LOM | TAA | VEN | FVG | LIG | EMR | TOS | UMB | MAR | LAZ | ABR | MOL | CAM | PUG | BAS | CAL | SIC | SAR |
| --- | --- | --- | --- | --- | --- | --- | --- | --- | --- | --- | --- | --- | --- | --- | --- | --- | --- | --- | --- | --- |
|  | (*n*=196) | (*n*=7) | (*n*=411) | (*n*=63) | (*n*=225) | (*n*=70) | (*n*=71) | (*n*=161) | (*n*=119) | (*n*=27) | (*n*=65) | (*n*=209) | (*n*=44) | (*n*=9) | (*n*=225) | (*n*=131) | (*n*=18) | (*n*=72) | (*n*=164) | (*n*=70) |
| Pneumococcal vaccine uptake | | | | | | | | | | |  |  |  |  |  |  |  |  |  |  |
| Yes, I did | 57 | 4 | 118 | 51 | 168 | 38 | 28 | 69 | 42 | 9 | 18 | 95 | 8 | 3 | 73 | 54 | 7 | 23 | 46 | 19 |
|  | (29%) | (57%) | (29%) | (81%) | (75%) | (54%) | (39%) | (43%) | (35%) | (33%) | (28%) | (45%) | (18%) | (33%) | (32%) | (41%) | (39%) | (32%) | (28%) | (27%) |
| No, but I would | 84 | 1 | 169 | 7 | 30 | 11 | 22 | 55 | 46 | 8 | 25 | 56 | 27 | 1 | 94 | 43 | 10 | 27 | 60 | 30 |
|  | (43%) | (14%) | (41%) | (11%) | (13%) | (16%) | (31%) | (34%) | (39%) | (30%) | (38%) | (27%) | (61%) | (11%) | (42%) | (33%) | (56%) | (38%) | (37%) | (43%) |
| No, and I would | 55 | 2 | 124 | 5 | 27 | 21 | 21 | 37 | 31 | 10 | 22 | 58 | 9 | 5 | 58 | 34 | 1 | 22 | 58 | 21 |
| not | (28%) | (29%) | (30%) | (8%) | (12%) | (30%) | (30%) | (23%) | (26%) | (37%) | (34%) | (28%) | (20%) | (56%) | (26%) | (26%) | (6%) | (31%) | (35%) | (30%) |
| Awareness of having priority for pneumococcal vaccination | | | | | | | | | | |  |  |  |  |  |  |  |  |  |  |
| Yes | 87 | 1 | 164 | 54 | 163 | 41 | 30 | 88 | 52 | 12 | 29 | 102 | 16 | 2 | 95 | 59 | 10 | 33 | 72 | 28 |
|  | (44%) | (14%) | (40%) | (86%) | (72%) | (59%) | (42%) | (55%) | (44%) | (44%) | (45%) | (49%) | (36%) | (22%) | (42%) | (45%) | (56%) | (46%) | (44%) | (40%) |
| No | 27 | 2 | 75 | 4 | 19 | 7 | 10 | 24 | 17 | 2 | 6 | 33 | 10 | 2 | 34 | 18 | 2 | 15 | 28 | 10 |
|  | (14%) | (29%) | (18%) | (6%) | (8%) | (10%) | (14%) | (15%) | (14%) | (7%) | (9%) | (16%) | (23%) | (22%) | (15%) | (14%) | (11%) | (21%) | (17%) | (14%) |
| Don’t know | 82 | 4 | 172 | 5 | 43 | 22 | 31 | 49 | 50 | 13 | 30 | 74 | 18 | 5 | 96 | 54 | 6 | 24 | 64 | 32 |
|  | (42%) | (57%) | (42%) | (8%) | (19%) | (31%) | (44%) | (30%) | (42%) | (48%) | (46%) | (35%) | (41%) | (56%) | (43%) | (41%) | (33%) | (33%) | (39%) | (46%) |
| Ease of access to get a pneumococcal vaccine | | | | | | | | | | |  |  |  |  |  |  |  |  |  |  |
| Very easy | 45 | 0 | 61 | 26 | 98 | 27 | 16 | 38 | 24 | 6 | 11 | 34 | 3 | 0 | 29 | 10 | 3 | 12 | 27 | 4 |
|  | (23%) | (0%) | (15%) | (41%) | (44%) | (39%) | (23%) | (24%) | (20%) | (22%) | (17%) | (16%) | (7%) | (0%) | (13%) | (8%) | (17%) | (17%) | (16%) | (6%) |
| Quite easy | 107 | 5 | 251 | 30 | 95 | 36 | 38 | 94 | 75 | 15 | 40 | 123 | 29 | 9 | 122 | 80 | 12 | 35 | 94 | 49 |
|  | (55%) | (71%) | (61%) | (48%) | (42%) | (51%) | (54%) | (58%) | (63%) | (56%) | (62%) | (59%) | (66%) | (100%) | (54%) | (61%) | (67%) | (49%) | (57%) | (70%) |
| Quite difficult | 35 | 1 | 81 | 4 | 14 | 6 | 15 | 21 | 17 | 6 | 12 | 43 | 10 | 0 | 52 | 36 | 2 | 18 | 38 | 14 |
|  | (18%) | (14%) | (20%) | (6%) | (6%) | (9%) | (21%) | (13%) | (14%) | (22%) | (18%) | (21%) | (23%) | (0%) | (23%) | (27%) | (11%) | (25%) | (23%) | (20%) |
| Very difficult | 9 | 1 | 18 | 3 | 18 | 1 | 2 | 8 | 3 | 0 | 2 | 9 | 2 | 0 | 22 | 5 | 1 | 7 | 5 | 3 |
|  | (5%) | (14%) | (4%) | (5%) | (8%) | (1%) | (3%) | (5%) | (3%) | (0%) | (3%) | (4%) | (5%) | (0%) | (10%) | (4%) | (6%) | (10%) | (3%) | (4%) |

*PIE*, Piedmont; *VDA*, Aosta Valley; *LOM*, Lombardy; *LIG*, Liguria; *TAA*, Trentino-South Tyrol; *VEN*, Veneto; *FVG*, Friuli-Venezia Giulia; *EMR*, Emilia-Romagna; *TOS*, Tuscany; *UMB*, Umbria; *MAR*, Marche; *LAZ*, Lazio; *ABR*, Abruzzo; *MOL*, Molise; *CAM*, Campania; *PUG*, Apulia; *BAS*, Basilicata; *CAL*, Calabria; *SIC*, Sicily; *SAR*, Sardinia.
