# Supplementary material for: Pneumococcal vaccine uptake among high-risk adults and children in Italy: results from the OBVIOUS project survey
Source: BMC Public Health. 2024 Mar 7;24:736. doi: 10.1186/s12889-024-18216-3 (PMC10921627; doi:10.1186/s12889-024-18216-3)
Supplement: Supplementary file 3 — Supplementary Material 3. [file 12889_2024_18216_MOESM3_ESM.docx]

## Additional Table 2. Uptake, awareness, worry, perception of safety, and ease of access among respondents with ≥65 years of age, overall and by gender.

| Characteristic | All | Males | Females |
| --- | --- | --- | --- |
|  | (*n* = 1321) | (*n* = 741) | (*n* = 580) |
| Pneumococcal vaccine uptake |  |  |  |
| Yes, I did | 445 (33.7%) | 274 (37.0%) | 171 (29.5%) |
| No, but I would | 490 (37.1%) | 274 (37.0%) | 216 (37.2%) |
| No, and I would not | 386 (29.2%) | 193 (26.0%) | 193 (33.3%) |
| Awareness of having higher priority for pneumococcal vaccination |  |  |  |
| Yes | 619 (46.9%) | 360 (48.6%) | 259 (44.7%) |
| No | 144 (10.9%) | 65 (8.8%) | 79 (13.6%) |
| Don’t know | 558 (42.2%) | 316 (42.6%) | 242 (41.7%) |
| Worry about getting sick with pneumococcal pneumonia |  |  |  |
| Not worried | 278 (21.0%) | 162 (21.9%) | 116 (20.0%) |
| A little worried | 689 (52.2%) | 382 (51.6%) | 307 (52.9%) |
| Quite worried | 289 (21.9%) | 156 (21.1%) | 133 (22.9%) |
| Very worried | 65 (4.9%) | 41 (5.5%) | 24 (4.1%) |
| Perception of the safety of pneumococcal vaccines |  |  |  |
| Very safe | 320 (24.2%) | 194 (26.2%) | 126 (21.7%) |
| Quite safe | 851 (64.4%) | 471 (63.6%) | 380 (65.5%) |
| Quite unsafe | 114 (8.6%) | 60 (8.1%) | 54 (9.3%) |
| Very unsafe | 36 (2.7%) | 16 (2.2%) | 20 (3.4%) |
| Perception of how easy it is to access healthcare facilities to get a pneumococcal vaccine |  |  |  |
| Very easy | 274 (20.7%) | 157 (21.2%) | 117 (20.2%) |
| Quite easy | 823 (62.3%) | 469 (63.3%) | 354 (61.0%) |
| Quite difficult | 185 (14.0%) | 98 (13.2%) | 87 (15.0%) |
| Very difficult | 39 (3.0%) | 17 (2.3%) | 22 (3.8%) |

*Notes:* Females include non-binary people.
