# Supplementary material for: Pneumococcal vaccine uptake among high-risk adults and children in Italy: results from the OBVIOUS project survey
Source: BMC Public Health. 2024 Mar 7;24:736. doi: 10.1186/s12889-024-18216-3 (PMC10921627; doi:10.1186/s12889-024-18216-3)
Supplement: Supplementary file 4 — Supplementary Material 4. [file 12889_2024_18216_MOESM4_ESM.docx]

## Additional Table 3. Uptake, awareness, worry, perception of safety, and ease of access among respondents with pneumopathy, overall and by gender.

| Characteristic | All | Males | Females |
| --- | --- | --- | --- |
|  | (*n* = 516) | (*n* = 268) | (*n* = 248) |
| Pneumococcal vaccine uptake |  |  |  |
| Yes, I did | 250 (48.4%) | 135 (50.4%) | 115 (46.4%) |
| No, but I would | 166 (32.2%) | 88 (32.8%) | 78 (31.5%) |
| No, and I would not | 100 (19.4%) | 45 (16.8%) | 55 (22.2%) |
| Awareness of having higher priority for pneumococcal vaccination |  |  |  |
| Yes | 275 (53.3%) | 148 (55.2%) | 127 (51.2%) |
| No | 92 (17.8%) | 49 (18.3%) | 43 (17.3%) |
| Don’t know | 149 (28.9%) | 71 (26.5%) | 78 (31.5%) |
| Worry about getting sick with pneumococcal pneumonia |  |  |  |
| Not worried | 83 (16.1%) | 41 (15.3%) | 42 (16.9%) |
| A little worried | 191 (37.0%) | 108 (40.3%) | 83 (33.5%) |
| Quite worried | 162 (31.4%) | 81 (30.2%) | 81 (32.7%) |
| Very worried | 80 (15.5%) | 38 (14.2%) | 42 (16.9%) |
| Perception of the safety of pneumococcal vaccines |  |  |  |
| Very safe | 137 (26.6%) | 77 (28.7%) | 60 (24.2%) |
| Quite safe | 275 (53.3%) | 137 (51.1%) | 138 (55.6%) |
| Quite unsafe | 75 (14.5%) | 38 (14.2%) | 37 (14.9%) |
| Very unsafe | 29 (5.6%) | 16 (6.0%) | 13 (5.2%) |
| Perception of how easy it is to access healthcare facilities to get a pneumococcal vaccine |  |  |  |
| Very easy | 81 (15.7%) | 46 (17.2%) | 35 (14.1%) |
| Quite easy | 244 (47.3%) | 130 (48.5%) | 114 (46.0%) |
| Quite difficult | 140 (27.1%) | 68 (25.4%) | 72 (29.0%) |
| Very difficult | 51 (9.9%) | 24 (9.0%) | 27 (10.9%) |

*Notes:* Females include non-binary people.
