# Supplementary material for: Pneumococcal vaccine uptake among high-risk adults and children in Italy: results from the OBVIOUS project survey
Source: BMC Public Health. 2024 Mar 7;24:736. doi: 10.1186/s12889-024-18216-3 (PMC10921627; doi:10.1186/s12889-024-18216-3)
Supplement: Supplementary file 5 — Supplementary Material 5. [file 12889_2024_18216_MOESM5_ESM.docx]

## Additional Table 4. Uptake, awareness, worry, perception of safety, and ease of access among respondents with cardiopathy, overall and by gender.

| Characteristic | All | Males | Females |
| --- | --- | --- | --- |
|  | (*n* = 575) | (*n* = 361) | (*n* = 214) |
| Pneumococcal vaccine uptake |  |  |  |
| Yes, I did | 268 (46.6%) | 178 (49.3%) | 90 (42.1%) |
| No, but I would | 189 (32.9%) | 120 (33.2%) | 69 (32.2%) |
| No, and I would not | 118 (20.5%) | 63 (17.5%) | 55 (25.7%) |
| Awareness of having higher priority for pneumococcal vaccination |  |  |  |
| Yes | 300 (52.2%) | 196 (54.3%) | 104 (48.6%) |
| No | 104 (18.1%) | 64 (17.7%) | 40 (18.7%) |
| Don’t know | 171 (29.7%) | 101 (28.0%) | 70 (32.7%) |
| Worry about getting sick with pneumococcal pneumonia |  |  |  |
| Not worried | 108 (18.8%) | 62 (17.2%) | 46 (21.5%) |
| A little worried | 232 (40.3%) | 156 (43.2%) | 76 (35.5%) |
| Quite worried | 161 (28.0%) | 103 (28.5%) | 58 (27.1%) |
| Very worried | 74 (12.9%) | 40 (11.1%) | 34 (15.9%) |
| Perception of the safety of pneumococcal vaccines |  |  |  |
| Very safe | 153 (26.6%) | 95 (26.3%) | 58 (27.1%) |
| Quite safe | 329 (57.2%) | 213 (59.0%) | 116 (54.2%) |
| Quite unsafe | 72 (12.5%) | 43 (11.9%) | 29 (13.6%) |
| Very unsafe | 21 (3.7%) | 10 (2.8%) | 11 (5.1%) |
| Perception of how easy it is to access healthcare facilities to get a pneumococcal vaccine |  |  |  |
| Very easy | 101 (17.6%) | 64 (17.7%) | 37 (17.3%) |
| Quite easy | 297 (51.7%) | 200 (55.4%) | 97 (45.3%) |
| Quite difficult | 124 (21.6%) | 73 (20.2%) | 51 (23.8%) |
| Very difficult | 53 (9.2%) | 24 (6.6%) | 29 (13.6%) |

*Notes:* Females include non-binary people.
