# Supplementary material for: Pneumococcal vaccine uptake among high-risk adults and children in Italy: results from the OBVIOUS project survey
Source: BMC Public Health. 2024 Mar 7;24:736. doi: 10.1186/s12889-024-18216-3 (PMC10921627; doi:10.1186/s12889-024-18216-3)
Supplement: Supplementary file 6 — Supplementary Material 6. [file 12889_2024_18216_MOESM6_ESM.docx]

**Additional Table 5.** Uptake, awareness, worry, perception of safety, and ease of access among respondents with diabetes, overall and by gender.

| Characteristic | All | Males | Females |
| --- | --- | --- | --- |
|  | (*n* = 750) | (*n* = 501) | (*n* = 249) |
| Pneumococcal vaccine uptake |  |  |  |
| Yes, I did | 403 (53.7%) | 296 (59.1%) | 107 (43.0%) |
| No, but I would | 204 (27.2%) | 125 (25.0%) | 79 (31.7%) |
| No, and I would not | 143 (19.1%) | 80 (16.0%) | 63 (25.3%) |
| Awareness of having higher priority for pneumococcal vaccination |  |  |  |
| Yes | 449 (59.9%) | 320 (63.9%) | 129 (51.8%) |
| No | 105 (14.0%) | 58 (11.6%) | 47 (18.9%) |
| Don’t know | 196 (26.1%) | 123 (24.6%) | 73 (29.3%) |
| Worry about getting sick with pneumococcal pneumonia |  |  |  |
| Not worried | 119 (15.9%) | 83 (16.6%) | 36 (14.5%) |
| A little worried | 283 (37.7%) | 176 (35.1%) | 107 (43.0%) |
| Quite worried | 190 (25.3%) | 118 (23.6%) | 72 (28.9%) |
| Very worried | 158 (21.1%) | 124 (24.8%) | 34 (13.7%) |
| Perception of the safety of pneumococcal vaccines |  |  |  |
| Very safe | 250 (33.3%) | 186 (37.1%) | 64 (25.7%) |
| Quite safe | 389 (51.9%) | 249 (49.7%) | 140 (56.2%) |
| Quite unsafe | 84 (11.2%) | 50 (10.0%) | 34 (13.7%) |
| Very unsafe | 27 (3.6%) | 16 (3.2%) | 11 (4.4%) |
| Perception of how easy it is to access healthcare facilities to get a pneumococcal vaccine |  |  |  |
| Very easy | 180 (24.0%) | 150 (29.9%) | 30 (12.0%) |
| Quite easy | 369 (49.2%) | 247 (49.3%) | 122 (49.0%) |
| Quite difficult | 137 (18.3%) | 75 (15.0%) | 62 (24.9%) |
| Very difficult | 64 (8.5%) | 29 (5.8%) | 35 (14.1%) |

*Notes:* Females include non-binary people.
